# Hydrogels for Neural Regeneration: Exploring New Horizons

**DOI:** 10.3390/ma17143472

**Published:** 2024-07-13

**Authors:** Hossein Omidian, Sumana Dey Chowdhury, Luigi X. Cubeddu

**Affiliations:** Barry and Judy Silverman College of Pharmacy, Nova Southeastern University, Fort Lauderdale, FL 33328, USA; sd2236@mynsu.nova.edu (S.D.C.); lcubeddu@nova.edu (L.X.C.)

**Keywords:** hydrogels, neural regeneration, neural scaffolding, clinical translation, biocompatibility

## Abstract

Nerve injury can significantly impair motor, sensory, and autonomic functions. Understanding nerve degeneration, particularly Wallerian degeneration, and the mechanisms of nerve regeneration is crucial for developing effective treatments. This manuscript reviews the use of advanced hydrogels that have been researched to enhance nerve regeneration. Hydrogels, due to their biocompatibility, tunable properties, and ability to create a supportive microenvironment, are being explored for their effectiveness in nerve repair. Various types of hydrogels, such as chitosan-, alginate-, collagen-, hyaluronic acid-, and peptide-based hydrogels, are discussed for their roles in promoting axonal growth, functional recovery, and myelination. Advanced formulations incorporating growth factors, bioactive molecules, and stem cells show significant promise in overcoming the limitations of traditional therapies. Despite these advancements, challenges in achieving robust and reliable nerve regeneration remain, necessitating ongoing research to optimize hydrogel-based interventions for neural regeneration.

## 1. Introduction

### 1.1. Neural Degeneration and Regeneration

Nerves are vital components of the peripheral nervous system, responsible for transmitting signals between the brain, spinal cord, and various parts of the body. Injury or damage to these nerves can result in a significant loss of motor, sensory, and autonomic functions, leading to conditions that profoundly affect quality of life [1]. Understanding the processes of nerve degeneration and regeneration is crucial for developing effective treatments to restore these functions.

Nerve degeneration occurs when axons, the long extensions of neurons, are damaged. This damage can be caused by various factors, including trauma, diseases like diabetes, and surgical procedures such as organ transplants. A common form of nerve degeneration is Wallerian degeneration (WD), which happens after the axon is severed. During WD, the distal part of the axon degenerates, creating a microenvironment that supports nerve regrowth [2]. Histological studies have shown that nerve degeneration is marked by the breakdown and absorption of myelin and axons, followed by the invasion of macrophages to clear debris [3].

Nerve regeneration is a complex process that involves the growth of new axons to replace those that have been damaged. This process is facilitated by Schwann cells, which respond to axonal injury by dedifferentiating, proliferating, and forming guiding structures called Bungner bands to direct regenerating axons [2]. However, successful nerve regeneration is not always achieved, and the quality of recovery can be affected by the extent and duration of degeneration prior to treatment [4].

Several conventional therapies have been explored to enhance nerve regeneration. These include the following:

- Pharmacological Treatments: Drugs like aldose reductase inhibitors and vasodilators have shown potential in enhancing nerve regeneration, particularly in conditions like diabetic neuropathy [5]. However, their efficacy needs thorough evaluation in terms of their ability to improve nerve regenerative capacity.
- Surgical Interventions: Traditional nerve conduits and nerve grafts are used to bridge gaps in damaged nerves. Studies have shown that the timing of these interventions is crucial, as delayed nerve repair can significantly compromise recovery [4].
- Electrical Stimulation (ES): Brief low-frequency ES has been demonstrated to accelerate Wallerian degeneration and promote nerve regeneration by enhancing the clearance of axonal and myelin debris and upregulating neurotrophic factors [6].
- Stem Cell Therapy: Stem cells offer a promising alternative for nerve regeneration. They can differentiate into Schwann-like cells and secrete neurotrophic factors, thereby promoting axonal growth and remyelination [7]. Various types of stem cells are being investigated for their potential in peripheral nerve regeneration.
- Hydrogels and Biomaterials: Innovative materials such as hydrogels are being explored to enhance nerve regeneration. These materials provide a supportive environment for nerve growth and can be used in combination with other treatments to improve outcomes [8,9,10,11,12,13,14,15].

Despite these advancements, many challenges remain in achieving robust and reliable nerve regeneration. The complexity of the molecular mechanisms involved and the need for precise control over the regeneration process continue to be significant hurdles. Ongoing research aims to develop therapies that not only promote axonal regrowth but also ensure the specificity and functionality of the regenerated nerves [16].

### 1.2. Hydrogels in Neural Regeneration

Hydrogels, composed of water-swollen polymer networks, have emerged as a promising solution in neural regeneration due to their biocompatibility, structural versatility, and ability to incorporate bioactive substances. These properties make them ideal for creating supportive microenvironments essential for nerve repair and functional recovery [17,18,19,20].

Polysaccharide hydrogels with internal scaffolds, for instance, show the potential for peripheral nerve regeneration [10,21,22]. Moreover, hydrogels combined with nanoparticles and neurotrophic factors have been investigated to promote nerve growth and improve functional recovery [23,24,25,26]. Injectable hydrogels are particularly noteworthy due to their exceptional properties, such as tunable mechanical properties and support for cell growth and tissue regeneration, making them suitable for various tissue engineering approaches, including cell encapsulation, the controlled release of therapeutic factors, and the incorporation of bioactive molecules.

Injectable hydrogels have demonstrated significant potential in treating central nervous system (CNS) injuries caused by ischemic stroke by providing supportive environments for cell growth and tissue regeneration [27]. They address issues like poor bioactivity and unstable drug release by enabling the controlled release of therapeutic agents [28,29,30,31]. Composite hydrogels incorporating materials like collagen, chitosan, and graphene oxide enhance effectiveness and stability, promoting Schwann cell proliferation and axon regrowth [32,33,34].

However, the application of hydrogels is not without challenges. Issues such as limited bioactivity, poor mechanical properties, and the need for specific structural configurations to support nerve regeneration must be addressed. Researchers are exploring various approaches, including electroconductive hydrogels, to enhance electrical properties and promote nerve repair [35,36,37]. Novel hydrogel compositions are designed to mimic the natural microenvironment of nervous tissue, providing localized trophic support and shielding neural cells from immune activity [38].

The versatility of hydrogel polymers, such as chitosan, alginate, collagen, hyaluronic acid, and peptides, is notable in neural applications:

- Chitosan-based Hydrogels: These are valued for their biodegradability and antimicrobial properties. Examples include thiolated chitosan hydrogels with taurine [8] and chitosan conduits with simvastatin-loaded Pluronic F-127 hydrogel [9]. Chitosan tubes prefilled with aligned fibrin nanofiber hydrogels (AFGs) [39] and chitosan/glycerol-beta-phosphate disodium salt hydrogels with Schwann cells [40] illustrate their versatility.
- Alginate-based Hydrogels: Known for their gel-forming capabilities and biocompatibility, these include formulations like alginate/chitosan hydrogels with 4-methylcatechol (4-MC) [22] and berberine [32], as well as alginate/gum arabic hydrogels with immobilized nerve growth factor (NGF) [24].
- Collagen-based Hydrogels: Leveraging properties of the natural extracellular matrix, these include chitosan/collagen hydrogel nerve conduits containing Schwann cells [11] and collagen type I hydrogels with naringin [30].
- Hyaluronic acid-based (HA) Hydrogels: Valued for promoting cell migration and proliferation, examples include injectable chitosan–hyaluronic acid hydrogels for the sustained release of NGF [41] and lithium chloride-loaded HA hydrogels [42].
- Peptide-based Hydrogels: These self-assembling hydrogels, like peptide amphiphile hydrogels delivering sonic hedgehog (SHH) protein [43] and neurotrophic peptide-functionalized hydrogels [44] provide customizable platforms for neural regeneration.

The regenerative capabilities of hydrogels are significantly enhanced by incorporating active ingredients, such as synthetic molecules, biomolecules, genes, and growth factors:

- Synthetic Molecules: Hydrogels incorporating simvastatin [9] and taurine [8] deliver targeted therapies promoting neurogenesis and reducing inflammation.
- Biomolecules: Bioactive molecules like 4-methylcatechol (4-MC) [22] and hesperidin [45] provide neuroprotective effects and support neural regeneration.
- Genes: Gene delivery via hydrogels is an innovative approach, with genetically modified cells overexpressing neurotrophic factors [46] enabling the sustained release of therapeutic genes.
- Growth Factors: The incorporation of nerve growth factor (NGF), brain-derived neurotrophic factor (BDNF), and fibroblast growth factor (FGF) into hydrogels [24,47,48,49] enhances their regenerative potential, supporting neuronal survival, axonal growth, and synaptic plasticity.

**Scheme 1:**
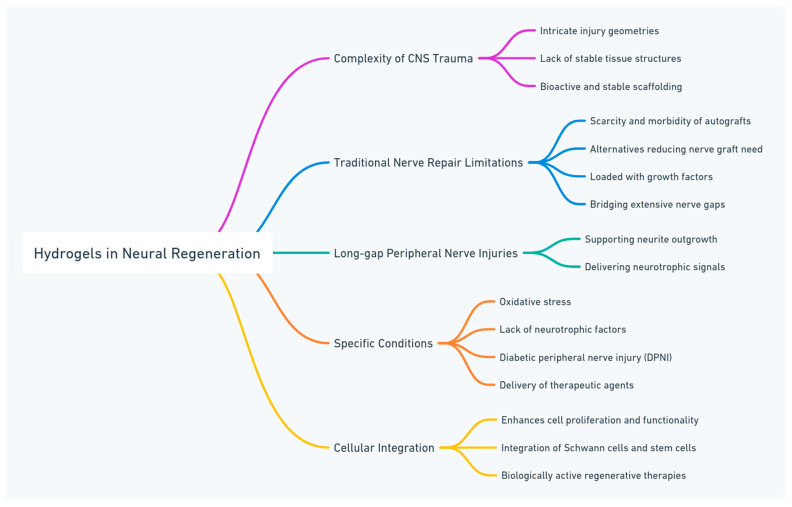
Potential uses and challenges of hydrogel interventions in neural regeneration.

In summary, while significant progress has been made in neural regeneration, the limitations of current treatments underscore the necessity for alternative interventions like hydrogels. These advanced biomaterials offer promising solutions to enhance nerve regeneration, address clinical challenges, and improve functional outcomes for patients with nerve injuries [9,34,39,47,50,51,52,53,54]. The combination of advanced hydrogel systems and active ingredients represents a promising strategy for developing effective treatments for neural injuries and degenerative diseases, offering new hope for improved clinical outcomes and enhanced quality of life (Scheme 1).

## 2. Chitosan-Based Hydrogels

This section discusses various chitosan-based hydrogels developed for nerve regeneration. The studies focus on different formulations, additives, and methodologies to enhance nerve repair. Evaluations include biocompatibility, morphological properties, and in vivo functionality. The common themes are chitosan’s favorable properties for biomedical use, yet each study’s unique approach highlights specific chemical or physical strategies for improved nerve regeneration.

Thiolated chitosan hydrogels containing varying concentrations of taurine were evaluated for their efficacy in peripheral nerve regeneration. The study assessed various morphological and biochemical properties, including pore size, swelling properties, weight loss, hemocompatibility, and cytocompatibility. Their in vivo functionality was tested using various methodologies. It was found that the hydrogel had an average pore size of 30–40 μm, with a weight loss of around 70% after seven days. The biocompatibility of the hydrogel was confirmed, and the addition of 1% taurine significantly enhanced sciatic nerve regeneration [8].

**Figure 1:**
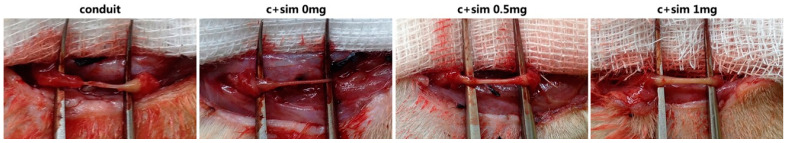
Gross views of regenerated sciatic nerves 10 weeks postoperatively. Regenerated sciatic nerves in rats with defects that were bridged by chitosan conduits filled with simvastatin/Pluronic F-127 hydrogel were much thicker than regenerated nerves in groups treated with hollow conduits or Pluronic F-127 hydrogel without simvastatin [9].

Chitosan conduits filled with simvastatin in Pluronic F-127 hydrogel were used to bridge 10 mm sciatic nerve defects in rats. The study analyzed the effects of different concentrations of simvastatin (0, 0.5, 1.0 mg) on nerve regeneration through various assessments, such as the sciatic functional index (SFI), electrophysiology, retrograde tracing, and histological and immunohistochemical analyses. The results indicated significant improvements in the SFI, compound muscle action potential peak amplitude, nerve conduction velocity, FG-labeled neurons, myelin sheath thickness, axon diameter, gastrocnemius wet weight, and muscle fiber area percentage and the increased expression of neurotrophic factors [9]. Figure 1 shows that regenerated nerves were observed spanning the defect gap in all four groups.

Chitosan tubes prefilled with an aligned fibrin nanofiber hydrogel (AFG), assembled via electrospinning and molecular self-assembly, were utilized to treat rabbit facial nerve defects. The study compared the compatibility of these hydrogels with Schwann cells and their ability to support axonal regeneration and remyelination against autologous nerve grafts and random fibrin nanofiber hydrogel. The AFG demonstrated enhanced adhesion, activity, and proliferation of Schwann cells, improved axonal regeneration, and superior functional recovery compared to the other methods, achieving results close to those of autologous nerve grafts [39].

A chitosan/glycerol-beta-phosphate disodium salt (CS/GP) hydrogel injected with Schwann cells was investigated for its potential in peripheral nerve regeneration. The study evaluated the gelation time and nerve regeneration using a rat sciatic nerve defect model, including electrophysiology, fluorogold retrograde tracing, histology, and muscle examination. The findings revealed that the CS/GP hydrogel alone impeded nerve regeneration, while the Schwann cell suspension group showed the best results, followed by the culture medium group. No regenerated nerves were observed in the hydrogel-injected groups, indicating that the CS/GP hydrogel was not effective, necessitating further research [40].

A carboxymethyl chitosan hydrogel manufactured through radiation-induced crosslinking was developed for nerve regeneration guides. The study focused on the degradation and crosslinking properties, physicochemical characteristics, cytotoxicity, in vivo reactions, and antimicrobial potential of hydrogel. The results indicated that 12% carboxymethyl chitosan (CMCS) aqueous solutions irradiated with an electron-beam dose of 25 kGy were suitable for the internal filling of biodegradable tubes. The hydrogel was nontoxic and showed antimicrobial activity against *E. coli*, and its hydrophilic properties make it a potential candidate for nerve regeneration guides [10].

Conductive black phosphorus nanosheets within a lipoic acid-modified chitosan hydrogel matrix incorporating tannic acid-modified black phosphorus nanosheets (BP@TA) and bicyclodextrin-conjugated tazarotene drug were evaluated for spinal cord injury repair. This injectable hydrogel exhibited enhanced conductivity, angiogenic potential, and motor function recovery. The study found that the hydrogel significantly facilitated angiogenesis and neurogenesis at the injury site, leading to notable improvements in motor function in a rat model [13].

Chitosan/beta-glycerophosphate/salt hydrogels with conductive aligned nanofibers composed of polycaprolactone, gelatin, and single-wall carbon nanotubes (SWCNTs) were developed for nerve regeneration. The study assessed the degradation rate, mechanical and electrical properties, interconnective structure, and biocompatibility of the hydrogels. The addition of conductive fibers improved axonal regrowth, provided suitable mechanical and electrical properties, and supported cell growth, making these hydrogels promising for nerve regeneration applications [55].

A substance-P-conjugated chitosan hydrochloride hydrogel (CSCl-SP) was evaluated for full-thickness wound healing. The stability of SP, as well as its effects on proliferation, migration, tube formation, angiogenesis-related gene and protein expression, extracellular matrix (ECM) deposition, and nerve regeneration, were analyzed. The hydrogel promoted proliferation, migration, and angiogenesis in vitro and enhanced vascularization, ECM deposition, and nerve regeneration in vivo. This led to the efficient recovery of full-thickness skin defects, highlighting the potential of CSCl-SP for regenerative medicine [21].

The research on chitosan-based hydrogels for neural regeneration includes a range of approaches, each exploring different formulations and additives to enhance nerve repair. Despite their varied methodologies, these studies share commonalities. A primary similarity across these studies is the use of chitosan as the fundamental component for the hydrogels, capitalizing on its favorable properties for biomedical applications, such as biocompatibility, biodegradability, and ease of chemical modification. Additionally, most studies involve evaluating the hydrogels’ biocompatibility and their effectiveness in promoting nerve regeneration, often through in vivo experiments using animal models [8,9,10,13,21,39,40,55].

The comparative analysis of chitosan-based hydrogels for nerve regeneration highlights that the most effective compositions combine chemical additives, physical structural enhancements, and cellular components. The inclusion of taurine [8] and simvastatin [9] has demonstrated significant improvements in nerve regeneration metrics, underscoring the value of chemical modifications. Additionally, the use of aligned fibrin nanofiber hydrogels [39] and conductive materials like single-wall carbon nanotubes [55] has been shown to enhance the physical and electrical properties of the hydrogels, thereby supporting axonal regrowth. The incorporation of Schwann cells further boosts regeneration outcomes, as seen in the study with chitosan/glycerol-beta-phosphate disodium salt hydrogel [40]. These strategies are justified by observed improvements in various nerve regeneration metrics, offering promising avenues for future research and application in the biomedical field.

Table 1 outlines different chitosan-based hydrogels researched for neural regeneration, highlighting their specific physical, chemical, and biological characteristics tailored to treat various neural diseases. Each hydrogel variant is designed to enhance nerve repair through properties such as biocompatibility, biodegradability, scaffold support, conductivity, and neurogenic potential.

## 3. Alginate-Based Hydrogels

This section explores alginate-based hydrogels for peripheral nerve and diabetic wound regeneration. Various studies have assessed hydrogels containing bioactive components like 4-MC, berberine, naringin, and hesperidin. Evaluations included pore size, biodegradability, cell proliferation, and hemocompatibility. The results showed enhanced nerve regeneration, biocompatibility, and controlled release properties, demonstrating the hydrogels’ potential in medical applications.

Alginate/chitosan hydrogels containing varying percentages of 4-methylcatechol (4-MC) were investigated for their potential in peripheral nerve regeneration. The study evaluated the pore size, biodegradability, hemocompatibility, and cytocompatibility of the hydrogels. The hydrogels demonstrated an appropriate pore size for cell proliferation of 26–42 µm and a biodegradability of 70% after seven days. The 10% 4-MC group showed enhanced sciatic nerve regeneration and improved PC12 cell proliferation, confirming the nontoxic nature of the hydrogels [22].

Chitosan/alginate hydrogels with berberine (Ber)-loaded chitosan nanoparticles and naringin (Nar)-loaded chitosan nanoparticles were also examined for their effects on peripheral nerve regeneration. The study focused on nanoparticle size, encapsulation efficiency, pore size, swelling, weight loss, compression strength, release profile, biocompatibility, and the proliferative effect on PC12 cells. The hydrogels exhibited suitable swelling, weight loss, and compression strength, were biocompatible, promoted PC12 cell proliferation, and enhanced sensory and motor function recovery along with anatomical healing in animal studies [23].

Another study explored alginate/chitosan hydrogels containing different dosages of hesperidin (0.1%, 1%, and 10% (*w*/*v*)) for peripheral nerve regeneration. Key parameters such as morphology, swelling properties, weight loss, hemocompatibility, cytocompatibility, porosity, biodegradability, and antibacterial properties were assessed. The hydrogels showed a porosity of 90% and a weight loss of 80% after 14 days. They demonstrated good blood compatibility and were nontoxic, with the 1% hesperidin group significantly improving the proliferation of olfactory ensheathing mesenchymal stem cells (OE-MSCs) and sciatic nerve regeneration [45].

**Figure 2:**
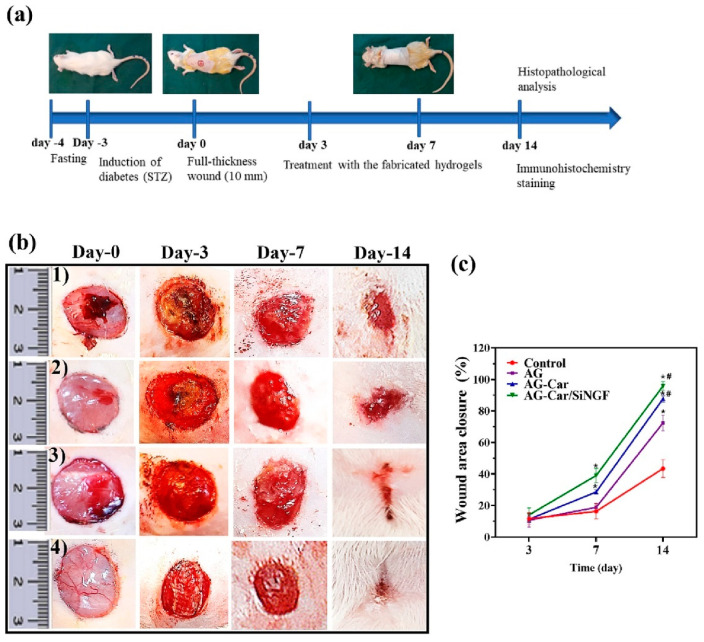
The evaluation of the wound-healing effects of hydrogels in STZ-induced diabetic rats. (**a**) A schematic illustration of the experiments. (**b**) The morphology of wounds treated with the hydrogels at different time points (0, 3, 7, and 14 days) during DFU healing. Untreated (control) group (1), AG hydrogel group (2), AG-Car hydrogel group (3), and AG-Car/SiNGF hydrogel group (4). (**c**) A graphical representation of the wound closure area measured using Image J software (V 1.8.0) (* *p* < 0.05, compared with control group; ^#^ *p* < 0.05, compared with control and AG hydrogel groups) [24].

Alginate/gum Arabic hydrogels enriched with immobilized nerve growth factor (NGF) in mesoporous silica nanoparticles (SiNGF) and carnosine (Car) were developed for diabetic wound regeneration. The study evaluated sustained NGF release, inflammation reduction, angiogenesis, re-epithelialization, collagen deposition, tissue neovascularization, TGF-beta expression, and nerve neurofilament presence. The hydrogel enabled controlled NGF release for over 21 days, reduced inflammation, increased angiogenesis, and improved re-epithelialization, collagen deposition, tissue neovascularization, transforming growth factor-beta (TGF-beta) expression, and nerve neurofilament presence in diabetic rat models [24]. Figure 2 illustrates the wound-healing effects of the hydrogels in Streptozotocin (STZ)-induced diabetic rats.

In a study of alginate/chitosan hydrogels containing different concentrations of berberine (0%, 0.1%, 1%, and 10% (*w*/*v*)), the effects on sciatic nerve regeneration were investigated. The study assessed structure, release profile, swelling, weight loss, cytocompatibility, hemocompatibility, pore size, and physical characterization, with a focus on the dose-dependent effect of berberine on cell proliferation. The initial pore size was about 39 μm, and the hydrogels exhibited 70% weight loss after 21 days. Hemocompatibility was confirmed, and a dose-dependent increase in cell proliferation was observed, with the 1% berberine group significantly enhancing sciatic nerve regeneration [32].

An alginate hydrogel scaffold mimicking the extracellular matrix (ECM) and loaded with melatonin was combined with a polycaprolactone outer layer to create a controlled-release microenvironment for peripheral nerve regeneration. The study examined the controlled release of melatonin, antioxidant properties, inflammation reduction, oxidative stress reduction, angiogenesis, and the ECM-like inner layer. The results indicated controlled melatonin release, an increased sciatic functional index, improved nerve electrical conduction, reduced inflammation and oxidative stress, enhanced angiogenesis, neurite extension, and axonal sprouting, and elevated fast-type myosin in the gastrocnemius muscle, effectively restoring the ECM-like microenvironment [28].

Alginate hydrogels incorporating magnetic short nanofibers (M.SNFs) made of wet-electrospun gelatin and superparamagnetic iron oxide nanoparticles (SPIONs) were used to encapsulate human olfactory mucosa stem cells (OE-MSCs). The study evaluated the storage modulus, cell encapsulation, bioactivity, neural-like differentiation, proliferation rate, viability, and the presence of SPIONs in the nanofibers. The hydrogels demonstrated a storage modulus within the range of nerve tissue, preserved cell viability after seven days, an enhanced proliferation rate in M.SNF/hydrogels, and accelerated neural-like differentiation of OE-MSCs due to the presence of SPIONs [29].

Studies on alginate-based hydrogels, much like those on their chitosan counterparts, share a common focus on biocompatibility, biodegradability, and the ability to support cell proliferation and nerve regeneration. The use of alginate is consistent across these studies, capitalizing on its natural origin, gelation properties, and compatibility with various bioactive components. Most studies also involve the incorporation of additional bioactive molecules or nanoparticles to enhance the regenerative capabilities of the hydrogels. Similarly, studies often employ animal models to evaluate the efficacy of these hydrogels in promoting peripheral nerve regeneration, assessing parameters like cell proliferation, functional recovery, and histological improvements [23,24,28,32,45].

When comparing these studies, the most effective alginate-based hydrogel compositions are those that integrate bioactive components, structural modifications, and suitable physical properties. The addition of 4-methylcatechol (4-MC) [22] and berberine [23,32] has shown significant improvements in nerve regeneration and cell proliferation, demonstrating the potential of these chemical modifications. Hydrogels enriched with NGF and carnosine [24], as well as melatonin-loaded scaffolds [28], enabled sustained release and antioxidant-rich environments that further enhanced tissue regeneration and nerve repair. Additionally, the inclusion of magnetic short nanofibers (SPIONs) [29] has been effective in enhancing neural-like differentiation and stem cell proliferation. These strategies, justified by observed improvements in various regenerative metrics, offer the most promising approaches for optimizing alginate-based hydrogels for medical applications. The combination of bioactive additives, structural enhancements, and controlled release properties ensures the best outcomes in nerve and wound regeneration.

Table 2 describes various alginate-based hydrogels designed for neural regeneration, highlighting their specific structural and functional characteristics tailored to treat neural diseases. These hydrogels are engineered to support nerve repair by offering features like biocompatibility, biodegradability, controlled release, enhanced angiogenesis, and the mimicking of the extracellular matrix.

## 4. Collagen-Based Hydrogels

This section discusses various studies on hydrogels used for nerve regeneration, highlighting alginate-based and collagen-based hydrogels. It focuses on their biocompatibility, bioabsorbability, mechanical strength, and scaffold support for cells. The studies explore different formulations and objectives, such as dual-layer designs, bioactive molecule incorporation, and advanced structural designs to enhance regenerative outcomes for peripheral nerve injuries.

A chitosan/collagen hydrogel nerve guidance conduit containing Schwann cells was developed for peripheral nerve regeneration. The study focused on the biocompatibility, mechanical strength, scaffold support for cells, bioabsorbability, and coaxial hydrogel layers of the conduit. The conduit, which was easily fabricated, demonstrated significant axonal extension of neurons and was both biocompatible and bioabsorbable while maintaining mechanical strength [11]. Another study expanded on this by encapsulating Schwann cells within a chitosan/collagen hydrogel nerve conduit in a rat sciatic nerve defect model. This design, with an outer chitosan hydrogel layer and an inner collagen hydrogel layer, facilitated superior motor functional recovery, axonal regrowth, and myelination compared to control groups, highlighting the positive early effects of Schwann cell-encapsulated conduits [12].

A collagen type I hydrogel containing chitosan nanoparticles loaded with insulin was examined for sciatic nerve regeneration. The study evaluated the proliferation rate of Schwann cells, sciatic functional index, hot-plate latency, compound muscle action potential amplitude, and wet gastrocnemius muscle weight. The results showed increased Schwann cell proliferation and significant improvements in the sciatic functional index, hot-plate latency, compound muscle action potential, and muscle weight, indicating the hydrogel’s potential for peripheral nerve regeneration [25]. Additionally, a hydroxyapatite nanoparticle-containing collagen type I hydrogel was explored for similar purposes. This hydrogel demonstrated significantly higher Schwann cell proliferation and enhanced functional behavior in rats, with an improved sciatic functional index, hot-plate latency, and muscle action potential amplitude compared to the collagen hydrogel and negative control groups [33].

In another study, a collagen type I hydrogel containing naringin was used for sciatic nerve regeneration. The study focused on the microstructure, swelling behavior, biodegradation, cyto/hemocompatibility, proliferation of Schwann cells, sciatic functional index, hot-plate latency, muscle action potential, and histopathologic examinations. The hydrogel had a porous structure with interconnected pores, had 70% weight loss after four weeks, and promoted higher Schwann cell proliferation. It significantly improved the sciatic functional index (22.13 ± 3.00 at 60 days post-implantation), hot-plate latency, and muscle action potential while reducing histological changes and resembling a normal nerve structure with an intact myelin sheath [30].

Nanofiber neural guidance channels (NGCs) containing a collagen hydrogel and 5% acetyl L-carnitine (ALC) were developed for nerve regeneration. The study assessed surface hydrophilicity, porosity, tensile strength, cell viability, cell attachment, and varying ALC concentrations (1%, 3%, 5%). The hydrogels with 5% ALC showed better cell viability and significant recovery potential in NGCs filled with ALC-containing hydrogel, improving histopathological and functional recovery in a sciatic nerve injury rat model [56]. Furthermore, elastic nerve guidance conduits (NGCs) prepared using a poly(lactide-co-caprolactone) (PLCL) membrane with a 3D-printed collagen hydrogel were investigated. These NGCs exhibited elasticity, a nanoporous structure, permeability, and microscale hydrogel patterning and were found to support superior functional recovery, axonal regeneration, and remyelination compared to the bulk collagen hydrogel, providing a favorable environment for nerve regeneration [57].

The research on collagen-based hydrogels for neural regeneration consistently shares several key similarities. Across different studies, collagen serves as the primary scaffold material due to its biocompatibility, bioabsorbability, and ability to support cell proliferation and tissue integration. Many of these hydrogels incorporate additional materials or bioactive molecules to enhance their regenerative capabilities. Moreover, these studies typically employ in vivo models, such as rat sciatic nerve defect models, to evaluate the hydrogels’ effectiveness in promoting nerve regeneration, assessing various parameters, like functional recovery, axonal regrowth, and muscle function [12,25,30,33,56,57].

The comparative analysis of collagen-based hydrogels for nerve regeneration suggests that the most effective compositions integrate bioactive molecules, advanced structural designs, and suitable physical properties. The incorporation of Schwann cells [11,12] has shown significant enhancements in motor functional recovery and axonal regrowth. Hydrogels with insulin-loaded chitosan nanoparticles [25] and hydroxyapatite nanoparticles [33] demonstrated substantial improvements in Schwann cell proliferation and various nerve regeneration metrics. Moreover, naringin-loaded collagen hydrogels [30] and nanofiber neural guidance channels with acetyl L-carnitine [56] provided notable enhancements in functional recovery and cell viability. The use of 3D-printed collagen hydrogel in elastic nerve guidance conduits [57] offered an advanced structural design that supports superior functional recovery and axonal regeneration. These strategies, adequately justified by observed improvements in nerve regeneration outcomes, offer the most promising avenues for optimizing collagen-based hydrogels for peripheral nerve injuries. The combination of bioactive additives, structural enhancements, and mechanical properties ensures the best outcomes in nerve regeneration applications.

Table 3 presents various collagen-based hydrogels engineered for neural regeneration, highlighting their structural and functional characteristics. These hydrogels are designed to support nerve repair by enhancing properties such as biocompatibility, biodegradability, mechanical strength, Schwann cell proliferation, and axonal regrowth, making them suitable for treating peripheral nerve injuries.

## 5. Hyaluronic Acid-Based Hydrogels

This section discusses various hyaluronic acid-based hydrogels developed for neural regeneration. These hydrogels are enhanced with bioactive molecules like NGF, BDNF, and anti-inflammatory agents to support nerve repair. Studies assess their biocompatibility, swelling, degradation, and functional recovery in animal models, highlighting differences in formulations, targeted injuries, and outcomes for peripheral and central nervous system repair.

An injectable chitosan/hyaluronic acid (CS-HA) hydrogel was designed for the sustained release of nerve growth factor (NGF) to aid in nerve regeneration. The study assessed the gelation time, interconnected channels, pore diameter, porosity, swelling behavior, degradation rate, NGF release profiles, biocompatibility, and the adhesion and proliferation of bone marrow-derived mesenchymal stem cells (BMMSCs). The CS-HA hydrogel gelled rapidly at pH 7.4, exhibited ~80% porosity with interconnected channels, and showed favorable swelling and degradation properties, with 70% degradation within eight weeks. It proved suitable for NGF release, had low cytotoxicity, and supported BMMSC adhesion and proliferation, making it a promising candidate for neural tissue engineering [41].

In another study, a hyaluronic acid hydrogel loaded with lithium chloride (LiCl) at a dose of 15 mEq and different doses of LiCl itself (2.5, 5, and 15 mEq) were investigated for nerve regeneration and motor function recovery. The study focused on biocompatibility, neuroprotective effects, anti-inflammatory effects, and histological and functional recovery measurements. The 5 mEq LiCl dose significantly increased nerve regeneration, positively impacted peripheral nerve injury recovery, improved motor function, and yielded favorable histological outcomes [42].

A hyaluronan/methylcellulose hydrogel modified with the anti-inflammatory peptide KAFAKLAARLYRKALARQLGVAA (KAFAK) and brain-derived neurotrophic factor (BDNF) was developed for spinal cord injury (SCI) regeneration. This minimally invasive hydrogel provided localized and sustained protein delivery, exhibiting anti-inflammatory effects, enhanced neuronal survival, and the proliferation of PC12 cells. The hydrogel reduced proinflammatory cytokines and glial scar formation while improving nerve tissue morphology and axonal regeneration in an SCI rat model, resulting in significant neurological function recovery [14].

An injectable hyaluronic acid/phenylboronic acid/poly(vinyl alcohol)/heparin hydrogel modified with cysteamine and phenylboronic acid and loaded with glial cell-derived neurotrophic factor (GDNF) was studied for peripheral nerve regeneration and pain relief. The hydrogel demonstrated biocompatibility, stability, antioxidative and anti-inflammatory properties, and the sustained release of GDNF. It improved sensorimotor function, prevented muscular atrophy, promoted nerve regeneration, reduced inflammation and oxidative stress, and alleviated pain. However, GDNF supplementation interrupted the recovery process, indicating the need for further investigation on the local administration of GDNF [58].

A hyaluronic acid-based hydrogel scaffold containing a matrix metalloproteinase-sensitive peptide, IKVAV (Ile- Lys-Val-Ala-Val) peptide, and brain-derived neurotrophic factor (BDNF) was explored for spinal cord regeneration. Human mesenchymal stem cells (hMSCs) were cultured in these hydrogels for 10 days to induce neuronal differentiation. This biomimetic hydrogel scaffold promoted biocompatibility, neural differentiation, and remodeling activity. The inclusion of an MMP-sensitive peptide crosslinker, IKVAV peptide from laminin, and BDNF resulted in the greatest improvement in locomotive tests in rats, enhanced the neuronal differentiation of human mesenchymal stem cells (hMSCs), and supported nerve regeneration in a spinal cord injury model. These findings suggested a favorable microenvironment for neural cell lineage differentiation and nerve regeneration [48].

A hyaluronic acid hydrogel functionalized with 2,2,6,6-tetramethylpiperidinyloxy (TEMPO) was studied for spinal cord transection recovery and bladder tissue protection. The hydrogel exhibited antioxidant properties, biocompatibility, and a porous structure and facilitated motor function restoration, tissue reconnection, and nerve fiber regeneration. It effectively protected bladder tissue from neurogenic damage, demonstrating a promising strategy for treating central nervous system diseases through antioxidant and lesion-bridging regulation [15].

In another study, a hyaluronic acid granular hydrogel nerve guidance conduit was evaluated for 10 mm long sciatic nerve gap regeneration in rats. The study simulated the extracellular matrix, promoting axonal extension, motor function recovery, electrophysiological function, and histological and morphological recovery. The granular hydrogel conduit achieved similar regeneration of sciatic nerve axons and myelin sheath and comparable recovery of electrophysiological and motor functions to autologous nerve transplantation, outperforming bulk hydrogel or silicone tube transplants [59].

The research on hyaluronic acid-based hydrogels for neural regeneration shares several key similarities. These hydrogels leverage hyaluronic acid’s natural properties, including its biocompatibility, biodegradability, and ability to support cell proliferation and tissue integration. Many studies incorporate additional bioactive molecules or modifications to enhance the regenerative potential of hydrogels. Most studies also employ in vivo models, such as rat sciatic nerve injury or spinal cord injury models, to assess the hydrogels’ effectiveness in promoting nerve regeneration and functional recovery [14,15,41,42,48,58,59].

The comparative analysis of hyaluronic acid-based hydrogels for neural regeneration suggests that the most effective compositions integrate bioactive molecules, anti-inflammatory agents, and advanced structural designs. The incorporation of NGF [41], BDNF [14,48], and lithium chloride [42] has shown significant enhancements in nerve regeneration and functional recovery. Hydrogels with anti-inflammatory peptides like KAFAK [14] and antioxidative agents such as TEMPO [15] provided notable improvements in neuronal survival, differentiation, and overall tissue recovery. Additionally, granular hydrogel conduits [59] and scaffolds with MMP-sensitive peptides and IKVAV [48] offered advanced structural designs that support superior functional recovery and neural differentiation. These strategies, adequately justified by observed improvements in various neural regeneration metrics, offer the most promising approaches for optimizing hyaluronic acid-based hydrogels for both peripheral and central nervous system repair. The combination of bioactive additives, structural enhancements, and targeted functionalization ensures the best outcomes in neural regeneration applications.

Table 4 outlines various hyaluronic acid-based hydrogels designed for neural regeneration, detailing their structural and functional characteristics. These hydrogels enhance nerve repair through properties such as biocompatibility, sustained release, anti-inflammatory and antioxidant effects, neuroprotection, and support for axonal extension, making them suitable for treating spinal cord and peripheral nerve injuries.

## 6. Peptide-Based Hydrogels

Peptide-based hydrogels are explored for nerve regeneration, mimicking the extracellular matrix and supporting cell growth. Studies on various hydrogels, including C16GSH, RADA16-I, and SF16, have shown their efficacy in promoting axonal growth, Schwann cell proliferation, and functional recovery. These hydrogels are biocompatible, often incorporating bioactive molecules to enhance regenerative capabilities, demonstrating their potential for treating different nerve injuries.

A peptide amphiphile hydrogel composed of C16GSH was evaluated for peripheral nerve regeneration compared to traditional collagen gels. It was able to promote Schwann cell proliferation and migration while mimicking the native extracellular matrix. Additionally, this hydrogel was found to be biocompatible, supported angiogenesis, and degraded over time without causing inflammation or an immune response, making it a suitable candidate for nerve regeneration applications [60].

Peptide amphiphile nanofiber hydrogels delivering the sonic hedgehog (SHH) protein were studied for cavernous nerve regeneration. This approach aimed to maintain neuronal integrity and prevent degeneration while supporting neuronal and glial signaling. The SHH treatment was successful in sustaining neuronal integrity, preventing degeneration, and promoting both retrograde and anterograde transport. This method showed significant potential as a regenerative therapy for cavernous nerve injury, particularly in preventing erectile dysfunction post-prostatectomy [43].

A synthesized nanofiber scaffold hydrogel, the peptide RADA16-I, was used for peripheral nerve regeneration in rats. This self-assembling peptide hydrogel demonstrated faster healing rates for sciatic nerve injuries compared to blank controls. Electrophysiological recordings showed improved nerve conduction velocities, indicating the hydrogel’s effective role in promoting sciatic nerve regeneration in the animal model [61].

Another study explored a neurotrophic peptide-functionalized self-assembling peptide nanofiber hydrogel (RAD/RGI) prefilled inside a hollow chitosan tube (hCST) to promote sciatic nerve regeneration. This hydrogel was functionalized with the neurotrophic peptide RGI, which indicated improved axonal regeneration, enhanced remyelination, and better motor functional recovery. Additionally, the hydrogel promoted PC12 cell adhesion, proliferation, and neuronal differentiation, creating a neurotrophic microenvironment conducive to effective peripheral nerve regeneration [44].

The RADA16-Mix hydrogel, a self-assembling peptide nanofiber hydrogel modified with IKVAV and RGD, was developed for sciatic nerve regeneration in rats. This hydrogel, designed to remain pH-neutral, induced greater axonal regeneration and Schwann cell migration, resulting in better functional recovery and the formation of new neuromuscular junction structures compared to the RADA16-I hydrogel [62].

For recurrent laryngeal nerve regeneration, the RADA16-I self-assembling peptide hydrogel was utilized in a rat model. The study evaluated neurite outgrowth, functional synapse formation, nerve regeneration, thyroarytenoid muscle atrophy, neurofilament-positive areas, and the presence of myelinated nerves. The RADA16-I hydrogel significantly increased the neurofilament-positive areas, the number of myelinated nerves, and the area of the thyroarytenoid muscle, demonstrating its effectiveness in promoting recurrent laryngeal nerve regeneration [63].

A BD PuraMatrix peptide hydrogel seeded with Schwann cells was investigated for sciatic nerve regeneration in rats. The hydrogel significantly increased the axonal regeneration distance and promoted the linear alignment of nerve fibers and Schwann cells. However, it did not show improvements in motoneuron regeneration or muscle recovery compared to nerve grafting [64].

Multidomain peptide (MDP) hydrogels, both anionic and cationic, were used as intraluminal fillers in electrospun poly(epsilon-caprolactone) (PCL) conduits for sciatic nerve regeneration in rats. The study examined the degradation time, electromyography results, gait analysis, histomorphometry, axon count, muscle weight retention, and functional recovery. PCL conduits filled with the anionic MDP showed better functional recovery, higher muscle action potential, greater muscle weight retention, and superior myelination compared to the cationic MDP, suggesting that the anionic MDP is a promising strategy for treating transected peripheral nerve injury [65].

A silk fibroin peptide (SF16) hydrogel scaffold was developed for peripheral nerve regeneration. In vitro tests on PC12 cells showed that the hydrogel supported cell viability and growth, and in vivo tests using a nerve gap model compared the SF16 hydrogel to physiological saline and collagen, evaluating recovery through walking track analysis and electrophysiological methods. The SF16 hydrogel scaffold significantly improved axonal regeneration and functional recovery and increased the axon density, axon diameter, and myelin thickness compared to physiological saline, supporting its potential for peripheral nerve injury repair [66].

Moreover, a human hair keratin hydrogel scaffold was studied for median nerve regeneration in nonhuman primates. A 1 cm nerve gap was grafted with a NeuraGen(R) collagen conduit and filled with a keratin hydrogel or saline. The research focused on electrophysiology, nerve histomorphometry, myofiber density, compound motor action potential (CMAP), and nerve conduction velocity (NCV). The keratin hydrogel-grafted nerves showed significant improvements in CMAP latency and NCV recovery, a larger nerve area, and a higher myofiber density compared to saline-treated nerves, enhancing peripheral nerve regeneration and motor recovery [67].

Peptide-based hydrogels for neural regeneration share several common features across the various studies. These hydrogels leverage the self-assembling properties of peptides to create a supportive extracellular-matrix-like environment conducive to cell growth and tissue regeneration. They are designed to be biocompatible, often incorporating additional bioactive molecules or peptides to enhance their regenerative capabilities. Most studies employ in vivo models, such as rat sciatic nerve injury or spinal cord injury models, to evaluate the effectiveness of these hydrogels in promoting nerve regeneration and functional recovery [44,60,61,62,63,64,65,66,67].

The comparative analysis of peptide-based hydrogels for nerve regeneration suggests that the most effective compositions integrate neurotrophic factors, cell adhesion molecules, and advanced structural designs. The incorporation of neurotrophic peptides like SHH [43], RGI [44], and IKVAV and RGD peptides [62] has shown significant enhancements in nerve regeneration and functional recovery. Hydrogels such as C16GSH [60] and SF16 [66] demonstrated substantial improvements in Schwann cell proliferation and axonal growth, indicating their potential for peripheral nerve injury repair. Additionally, the use of RADA16-I for specific nerve injuries like recurrent laryngeal nerve regeneration [63] and the anionic MDP hydrogels in PCL conduits [65] provided notable improvements in functional recovery and myelination. The combination of bioactive additives, structural enhancements, and suitable physical properties ensures the best outcomes in nerve regeneration applications. These strategies, adequately justified by observed improvements in various nerve regeneration metrics, offer the most promising approaches to optimizing peptide-based hydrogels for neural tissue engineering and repair.

Table 5 summarizes various peptide-based hydrogels designed for neural regeneration, detailing their physical, chemical, and biological properties. These hydrogels are engineered to support nerve repair through features like biocompatibility, enhanced Schwann cell proliferation, axonal regeneration, and neuronal differentiation, making them suitable for treating peripheral and specific nerve injuries.

## 7. Hydrogels with Specific Growth Factors and Cells

This section details various studies on hydrogels containing specific growth factors and cells for neural regeneration. It highlights different formulations and objectives aimed at promoting spinal cord and peripheral nerve regeneration. Utilizing biocompatible materials and stem cells, these hydrogels show potential in enhancing nerve regeneration and functional recovery in rodent models, targeting diverse neural injuries with tailored therapeutic goals.

An injectable thermosensitive hydrogel composed of chitosan/beta-glycerophosphate/hydroxyethyl cellulose (CS/beta-GP/HEC) encapsulating nerve growth factor (NGF)-overexpressing human adipose-derived mesenchymal stem cells (hADSCs) was studied for spinal cord regeneration in rats. The CS/beta-GP/HEC hydrogel with NGF-overexpressing hADSCs significantly promoted cell proliferation, reduced the cavity size at the spinal cord injury site, and improved locomotor functions, demonstrating its effectiveness in spinal cord regeneration [68].

A chitosan/beta-glycerophosphate (C/GP) hydrogel containing nerve growth factor (NGF) was developed for facial nerve regeneration in rats. The study examined drug delivery, scaffold properties, electrophysiological assessment, vibrissae movement, and morphological analysis of a rat facial nerve. The C/GP-NGF hydrogel used in an autologous vein conduit showed recovery rates of vibrissae movement and compound muscle action potentials (CMAPs) comparable to autologous implantation. Additionally, it resulted in larger regenerated axons and thicker myelin sheaths compared to the NGF solution alone, indicating improved facial nerve regeneration [49].

A hydrogel composed of hyaluronic acid and laminin (NVR-Gel) filled with Schwann cells (SCs) genetically modified to overexpress glial cell line-derived neurotrophic factor (GDNF) or fibroblast growth factor 2 (FGF-2 (18 kDa)) was examined for peripheral nerve regeneration. The study assessed neurotrophic factor production, neurite outgrowth, Schwann cell viability, axonal outgrowth, and sensory and motor regeneration. Chitosan/NVR-Gel/SC nerve guides with FGF-2 (18 kDa)-overexpressing SCs significantly improved axonal outgrowth and functional regeneration, overcoming initial obstacles encountered with NVR-Gel alone [46].

**Figure 3:**
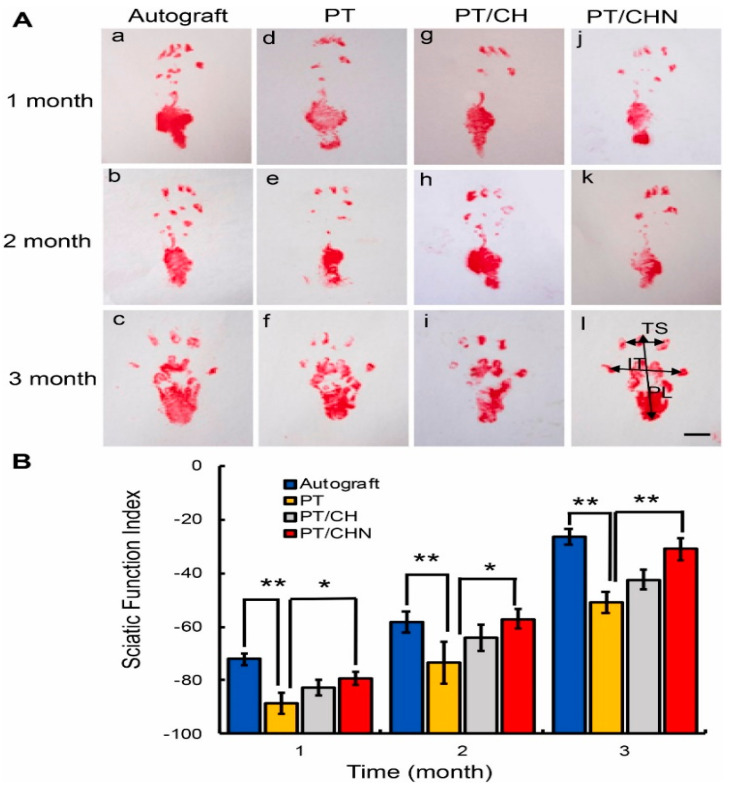
Walking track analyses in vivo. (**A**): Footprint collected on the walking track at different time points (1, 2, and 3 months after implantation): first column: autograft group; second column: PT group; third column: PT/CH group; fourth column: PT/CHN group. Scale bar, 5 mm. (**B**): SFIs calculated at different time points (1, 2, 3 months after implantation) * *p* < 0.05, ** *p* < 0.01 [26].

Poly(D,L-lactic acid) (PDLLA)/β-tricalcium phosphate (β-TCP) nerve conduits filled with injectable chitosan/hyaluronic acid hydrogel featuring the sustained release of nerve growth factor (NGF) were evaluated for peripheral nerve regeneration. This bioconjugated hydrogel was assessed for porosity; mass loss; NGF release; neuronal cell adhesion, spreading, and differentiation; and axon regeneration and myelination. The results indicated that PDLLA/β-TCP nerve conduits filled with CS-HA/NGF hydrogel significantly improved axon regeneration, myelination, and functional recovery in a 10 mm sciatic nerve defect rat model, highlighting its potential in neural tissue engineering [26]. Figure 3 illustrates the assessment of functional recovery in all surgically operated animals by analyzing the footprints of their right paws and calculating the sciatic nerve indices at each time point.

Poly(2-hydroxyethyl methacrylate-co-methyl methacrylate) (PHEMA-MMA) tubes filled with collagen gel impregnated with neurotropin-3, brain-derived neurotrophic factor (BDNF), or acidic fibroblast growth factor (FGF-1) were studied for peripheral nerve regeneration in rats. The study focused on biostability, biocompatibility, growth factor impregnation, histomorphometric analysis, nerve regeneration, and functional outcomes. PHEMA-MMA tubes with FGF-1 showed nerve regeneration outcomes comparable to autografts and significantly better than tubes with collagen alone, supporting their potential as an alternative to autografts for nerve injury repair [47].

Hierarchically aligned fibrin hydrogel microfibers laden with mesenchymal stem cells (MSCs) were used for spinal cord transection injury repair. This study examined MSC neural differentiation, nerve fiber regeneration, the aligned fiber structure, GAP-43 and NF-positive nerve fibers, and motor function recovery. The MSC-laden microfibers enhanced neural differentiation, significantly promoted nerve fiber regeneration, and improved motor function and electrophysiological expression, demonstrating the potential of MSC-laden microfibers for spinal cord injury repair [50].

An erythropoietin (EPO)-loaded multifunctional hydrogel combined with adipose-derived stem cells (ADSCs) was evaluated for neurogenic erectile function recovery. The hydrogel significantly improved erectile function, enhanced neural differentiation, preserved the vascular endothelium, prevented penile fibrosis, and accelerated Schwann cell migration, showing the potential for clinical translation in treating neurogenic erectile dysfunction [69].

A Poloxamer hydrogel delivering adipose-derived stem cells (ASCs) was investigated for peripheral nerve regeneration in rats. The study focused on ASC viability, axonal regrowth, reinnervation of muscle tissue, immunostaining, qPCR, and histological analysis. The hydrogel facilitated the longest axonal regrowth and improved the expression of factors aiding muscle reinnervation, suggesting a promising therapeutic approach for addressing multiple facets of nerve and muscle unit regeneration [70].

A heparin/Poloxamer thermosensitive hydrogel co-delivered with basic fibroblast growth factor (bFGF) and nerve growth factor (NGF) was evaluated for peripheral nerve regeneration in diabetic rats. The hydrogel significantly facilitated Schwann cell proliferation and enhanced axonal regeneration, remyelination, and motor function recovery, associated with the activation of the PI3K/Akt, JAK/STAT3, and MAPK/ERK signaling pathways, providing a promising therapeutic option for peripheral nerve regeneration in diabetic patients [71].

A decellularized porcine nerve-derived hydrogel filler peripheral nerve matrix (PNM) was used within conduits for nerve gap repair in rats. The study focused on nerve-specific matrix components, nerve growth factors, gait analysis, electrophysiology, and axon count. The PNM within conduits improved electrophysiologic responses and axon counts compared to empty conduit controls, supporting functional recovery for up to 24 weeks post-injury, indicating its potential benefits for treating nerve gap injuries [72].

A decellularized nerve matrix hydrogel was derived from a porcine sciatic nerve (pDNM-G) with longitudinally oriented microchannels through unidirectional freeze-drying, creating A-pDNM-G scaffolds for peripheral nerve regeneration. The a-pDNM-G scaffolds significantly promoted neurite extension, Schwann cell migration, axonal extension, and functional recovery in 15 mm rat sciatic nerve defects, with further improvements observed by incorporating nerve growth factor [73].

A fibrin hydrogel combined with poly(lactic-co-glycolic) acid (PLGA) microspheres encapsulating tacrolimus, along with rat adipose-derived mesenchymal stem cells (MSCs), was examined for peripheral nerve regeneration. This study focused on tacrolimus encapsulation, sustained release, MSC viability, and cytotoxicity. The combined delivery of 100 ng/mL tacrolimus and MSCs in the fibrin hydrogel showed no cytotoxic effects, maintained stem cell viability, and suggested the potential for enhancing peripheral nerve regeneration [74].

Moreover, a biodegradable gelatin hydrogel (Medgel) containing olfactory stem cells (OSCs) harvested from newborn mice was studied for facial nerve regeneration in mice. The research assessed OSC isolation, neural stem cell markers, trophic support, growth factors, Medgel retention, nerve regeneration, and peripheral nerve function. OSCs with Medgel accelerated recovery from facial nerve palsy, enhanced peripheral nerve function, and increased the number of regenerated nerve fibers, suggesting a promising treatment option for facial nerve injury recovery [75].

Hydrogels incorporating specific growth factors and cells for neural regeneration share several common features. They utilize biocompatible and biodegradable materials like chitosan, hyaluronic acid, and various synthetic polymers to create scaffolds that support cell proliferation and tissue integration. These hydrogels often encapsulate growth factors such as nerve growth factor (NGF), brain-derived neurotrophic factor (BDNF), and fibroblast growth factors (FGFs), as well as stem cells like mesenchymal stem cells (MSCs), Schwann cells, and adipose-derived stem cells (ASCs), to enhance their regenerative capabilities. Most studies employ in vivo models, typically involving rodent nerve injury models, to evaluate the hydrogels’ effectiveness in promoting nerve regeneration and functional recovery [26,46,47,49,50,68,69,70,71,72,73,74,75].

The comparative analysis of hydrogels with specific growth factors and cells for neural regeneration suggests that the most effective compositions integrate neurotrophic factors, stem cells, and advanced structural designs. The incorporation of NGF-overexpressing hADSCs [68], Schwann cells overexpressing FGF-2 (18 kDa) [46], and various growth factors like NGF, BDNF, and FGF-1 [47] has shown significant enhancements in nerve regeneration and functional recovery. Hydrogels such as C/GP-NGF [49] and CS-HA/NGF [26] demonstrated substantial improvements in axonal growth and myelination, indicating their potential for peripheral nerve injury repair. Additionally, the use of aligned fibrin hydrogel microfibers with MSCs [50] and the combination of Poloxamer with ASCs [70] provided notable improvements in neural differentiation and functional recovery.

Table 6 outlines various hydrogels loaded with specific growth factors and cells, designed for neural regeneration. These hydrogels enhance nerve repair through the sustained release of growth factors, support for cell adhesion and proliferation, and improved functional recovery, making them suitable for treating various neural injuries, including peripheral nerve and spinal cord injuries.

## 8. Hydrogels with Conductive Properties

This section discusses the development of conductive hydrogels for peripheral nerve regeneration. Various formulations, including a chitosan/aniline pentamer, graphene oxide composites, and polypyrrole-based hydrogels, are characterized by properties like electroactivity, biocompatibility, and self-healing. These hydrogels promote nerve cell growth, repair nerve defects, and enhance functional recovery, demonstrating significant potential in neural tissue engineering.

An electroactive chitosan/aniline pentamer hydrogel (CS-AP) was developed for peripheral nerve regeneration. This hydrogel was characterized by its electroactivity, degradation, gelation time, tensile strength, conductivity, cytocompatibility, capillary formation, nerve defect repair, and muscle reinnervation. The CS-AP10 hydrogel induced significant capillary formation, repaired sciatic nerve defects, and enhanced the reinnervation of the gastrocnemius muscle compared to other groups, demonstrating its potential for peripheral nerve injury regeneration and functional recovery [76].

A chitosan/oxidized hydroxyethyl cellulose/reduced graphene oxide/asiaticoside liposome hydrogel was created for nerve regeneration. The hydrogel was nontoxic, and its conductivity was 5.27 ± 0.42 × 10^−4^ S/cm. This hydrogel promoted the differentiation and proliferation of nerve cells, significantly inhibited scar formation, provided continuous asiaticoside release, and enhanced peripheral nerve function recovery, making it a promising candidate for nerve regeneration [51].

A conductive sodium alginate/carboxymethyl chitosan hydrogel doped with polypyrrole (SA/CMCS/PPy) was investigated for peripheral nerve regeneration. The SA/CMCS/PPy hydrogel showed good biocompatibility, and its conductivity ranged from 2.41 × 10^−5^ to 8.03 × 10^−3^ S cm^−1^. It also enhanced cell growth with electrical stimulation and effectively promoted nerve regeneration in an animal model, demonstrating its potential in neural tissue engineering [77].

A plasticine-like conductive hydrogel consisting of gelatin, polypyrrole, and tannic acid (GPT) was studied for peripheral nerve regeneration. The GPT hydrogel exhibited self-healing properties, electrical conductivity, and tunable polypyrrole concentration. In vitro and in vivo studies showed that this hydrogel promoted axonal regeneration and remyelination, prevented denervation atrophy, and enhanced functional recovery, suggesting its potential for clinical application in peripheral nerve injury repair [52].

Conductive graphene oxide/oligo(polyethylene glycol fumarate) (GO-OPF) hydrogel composites with a functionalized surface were evaluated for nerve regeneration. The study examined the hydrogels’ electrical conductivity, chemical and thermal properties, biocompatibility, nerve cell adhesion, proliferation, and differentiation. The incorporation of graphene oxide acrylate (GOA) sheets into OPF hydrogels enhanced nerve cell adhesion, proliferation, and neurite extension, supporting their potential application in nerve regeneration, particularly with additional functional groups to improve cell activities [78].

Reduced graphene oxide/gelatin-methacrylate (r(GO/GelMA)) hydrogel nerve guidance conduits (NGCs) were assessed for peripheral nerve regeneration. The study focused on the hydrogels’ electrical conductivity, flexibility, mechanical stability, permeability, neuritogenesis, muscle weight, electroconduction velocity, and sciatic nerve function index. The r(GO/GelMA) NGCs significantly enhanced nerve regeneration, as indicated by improved muscle weight, electroconduction velocity, and the sciatic nerve function index in a rat model, demonstrating the feasibility of electrically conductive hydrogel NGCs as functional conduits for improved nerve regeneration in preclinical studies [53].

A zwitterionic conductive hydrogel fabricated by the copolymerization of sulfobetain methacrylate and hydroxyethyl methacrylate was developed as a nerve guidance conduit for peripheral nerve regeneration in rats. This hydrogel was characterized by mechanical stability, electrical conductivity, cytocompatibility, Schwann cell proliferation, neurite length, and regeneration microenvironment. The zwitterionic hydrogel implanted into a 10 mm sciatic nerve defect rat model facilitated efficient peripheral nerve regeneration, with electrophysiological and morphological analysis showing regeneration effects similar to autologous nerve transplantation, enhancing Schwann cell growth and neurite extension [79].

**Figure 4:**
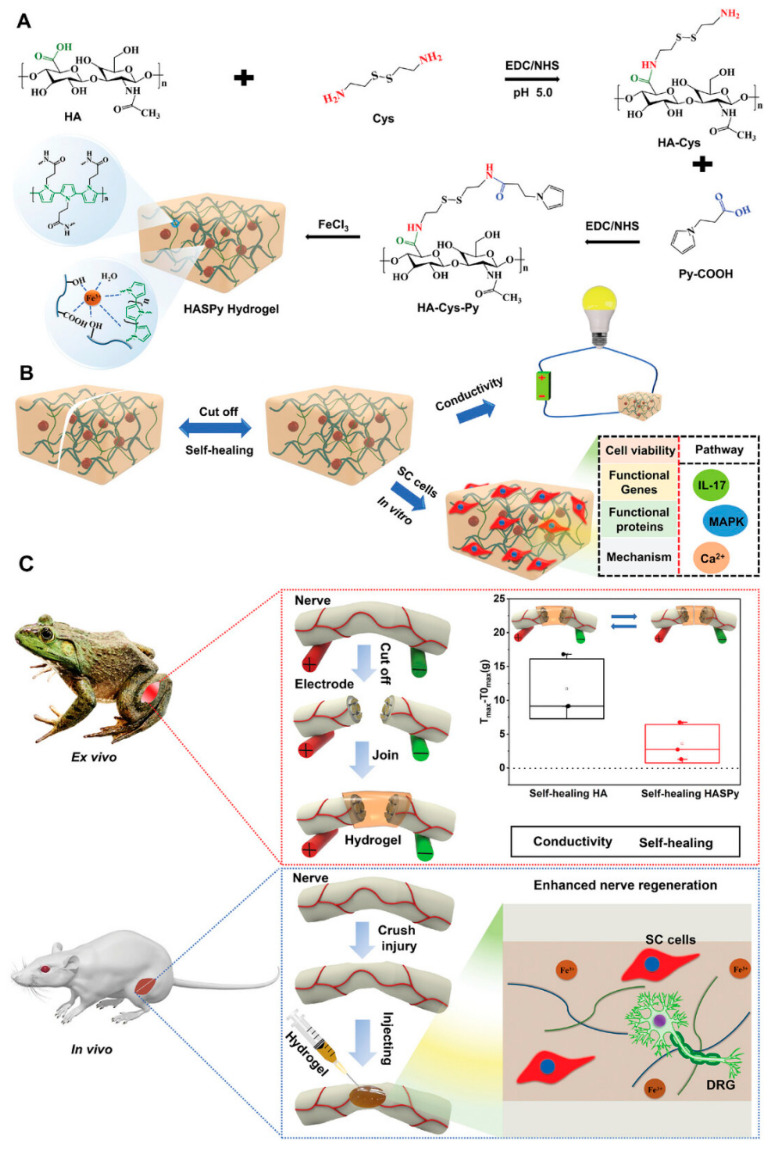
(**A**) A schematic diagram of the preparation of an injectable self-healing conductive hydrogel. (**B**) A schematic of the self-healing and electrical conductivity of the hydrogel and its regulation of the expression of Schwann functional genes and proteins. (**C**) A schematic representation of the bullfrog sciatic nerve placed in the hydrogel ex vivo and the rat peripheral nerve regenerated in vivo by injection [35].

A self-healing electroconductive hydrogel (HASPy) made from hyaluronic acid (HA), cystamine (Cys), and pyrrole-1-propionic acid (Py-COOH) was investigated for promoting peripheral nerve regeneration. This hydrogel exhibited self-healing properties, injectability, biodegradability, and biocompatibility and activated the IL-17 signaling pathway, enhancing Schwann cell myelination. The HASPy hydrogel promoted functional recovery and remyelination in a rat sciatic nerve injury model, providing insights into cell–matrix interactions and its potential use as an advanced scaffold for neural regeneration [35]. Figure 4 provides a schematic representation of the HASPy hydrogel’s preparation and application process.

An injectable, self-healing, conductive hydrogel (ACCP) was created by grafting polyaniline (PANI) onto carboxymethyl chitosan (CMCS) and crosslinking with aldehyde-based hyaluronic acid (ALHA). This hydrogel was designed for peripheral nerve regeneration and motor functional recovery. The ACCP hydrogel demonstrated conductivity, self-healing, injectability, a high elastic modulus, and biocompatibility, enhanced nerve conduction velocity, decreased the sciatic nerve functional index, induced axon extension and remyelination, and prevented muscle denervation, suggesting its potential for clinical application in peripheral nerve injury treatment [36].

Adhesive conductive immunomodulatory nerve hydrogel bandages were prepared from extracellular matrix (ECM), oxidized polysaccharides, and poly(3,4-ethylenedioxythiophene)/poly(styrenesulfonate) (PEDOT/PSS). These bandages were developed for nerve regeneration. The hydrogel bandages promoted nerve regeneration, enabled anatomical and functional recovery, prevented muscle atrophy, and formed a stable electrical bridge with electroresponsive neural tissue, suggesting critical clinical applications in peripheral nerve regeneration [37].

Conductive hydrogel scaffolds with in situ electrical generation capability, combining conductive hydrogel and wireless power transmitter, were developed for nerve regeneration. This innovative approach utilized wireless electrical stimulation via capacitive coupling and electrostatic induction, supporting functional recovery and neural tissue repair in a spinal cord injury model. The conductive hydrogel promoted remyelination, accelerated axon regeneration, and facilitated endogenous neural stem cell differentiation, showing promise in noninvasive, adjustable electrical stimulation for translational medicine [80].

A black phosphorus (BP) hydrogel loaded with neuregulin 1 (Nrg1) was studied for peripheral nerve regeneration. The BP hydrogel nerve guidance conduits (NGCs) were characterized by flexibility, nerve regeneration-related cell induction, Schwann cell proliferation, neuron-branch elongation, and axon remyelination. In vivo immunofluorescence studies showed that BP hydrogel NGCs loaded with Nrg1 promoted sciatic nerve regeneration and axon remyelination, demonstrating their potential as a treatment for peripheral nerve injuries [81].

A biomimetic silk fibroin hydrogel incorporating graphene oxide and fibroblast exosomes was developed for nerve regeneration. This conductive hydrogel was assessed for its conductivity, electron transmission, cellular behavior modulation, axon and myelin regeneration, and vascular regeneration. The incorporation of fibroblast exosomes enhanced axon and myelin regeneration, facilitated vascular regeneration via the VEGF/NOTCH signaling pathway, and led to substantial functional recovery in a rat sciatic nerve transection model [54].

Synthetic hydrogels with conductive properties for neural regeneration exhibit several common characteristics. These hydrogels are designed to be biocompatible and biodegradable, providing a supportive environment for nerve regeneration. They incorporate conductive materials to enhance electrical conductivity and promote cell proliferation, differentiation, and axonal growth. Additionally, many studies combine these hydrogels with bioactive molecules to further support nerve repair and functional recovery. Most of these studies employ in vivo models, typically using rodents, to evaluate the hydrogels’ effectiveness in promoting nerve regeneration and functional recovery [35,37,52,53,54,76,77,79,80,81].

The comparative analysis of conductive hydrogels for neural regeneration suggests that the most effective compositions integrate conductive materials, bioactive molecules, and advanced structural designs. The incorporation of polypyrrole, graphene oxide, and other conductive polymers [51,53] has shown significant enhancements in nerve regeneration and functional recovery. Hydrogels with self-healing properties, such as GPT [52] and HASPy [35], demonstrated substantial improvements in axonal growth and remyelination, indicating their potential for peripheral nerve injury repair. Additionally, the use of bioactive molecules like neuregulin 1 (Nrg1) in black phosphorus hydrogels [81] and fibroblast exosomes in silk fibroin hydrogels [54] provided notable improvements in neural differentiation and functional recovery.

Table 7 describes various synthetic hydrogels with conductive properties engineered for neural regeneration. These hydrogels support nerve repair through features like biocompatibility, electrical conductivity, enhanced cell proliferation, and remyelination, making them suitable for treating peripheral nerve and spinal cord injuries.

## 9. Various Synthetic and Composite Hydrogels

This section explores various synthetic and composite hydrogels designed for nerve regeneration. It covers different hydrogel compositions, including graphene oxide, collagen/chitosan, polyacrylamide/chitosan, and more, emphasizing their properties, fabrication methods, and effectiveness in enhancing axon growth, myelination, and functional recovery in various nerve injury models. The studies highlight the potential of these hydrogels as alternatives to traditional nerve repair techniques.

Silicone conduits filled with ammonia-functionalized graphene oxide (NH_2_-GO) and frankincense (Fr) embedded in collagen/chitosan hydrogel were evaluated for nerve regeneration. This study investigated neuroregenerative compounds, graphene oxide, frankincense, collagen/chitosan hydrogel, nerve regeneration, and myelin synthesis. The combination of NH_2_-GO and Fr resulted in an increased number of regenerated axons and thicker myelin sheaths, suggesting a synergistic effect on axon regrowth and myelin regeneration in a facial nerve injury model [82].

Polyacrylamide/chitosan (PAM/CS) composite hydrogels with elasticity and topographical guidance were designed for nerve regeneration. These hydrogels were created using in situ free-radical polymerization and micro-molding, focusing on elasticity, topographical guidance, neurite growth, and axon adhesion and extension. The PAM/CS composite hydrogel with an elastic modulus of 5.822 kPa/8.41 kPa and groove width of 30 µm promoted strong neurite growth, better-oriented status, and effective peripheral nerve regeneration in a rabbit sciatic nerve defect model, suggesting a new strategy for nerve injury treatment [83].

Collagen crosslinked with poly(N-isopropylacrylamide) (PNiPAAm) terpolymer scaffolds grafted with laminin pentapeptide YIGSR was studied for nerve regeneration. These bioactive hydrogel-filament scaffolds incorporated supporting fibers to enhance neurite extension, directional growth, and nerve regeneration. The inclusion of supporting fibers in the collagen/TERP scaffolds significantly increased neurite extension and influenced neurite morphology and function, demonstrating the potential value of these hybrid scaffolds as conduits in peripheral nerve repair [84].

A polyacrylonitrile (PAN) conduit filled with a fibrin hydrogel and graphene quantum dots (GQDs), incorporating Wharton’s jelly-derived mesenchymal stem cells (WJMSCs) differentiated into Schwann cells, was developed for sciatic nerve injury. The study focused on the controlled diameter and size of the conduit, Schwann cell differentiation, integration with the fibrin gel and lumen, and the role of graphene quantum dots in enhancing conductivity and cell support. This composite hydrogel-filled PAN conduit with GQDs showed significantly higher sensorial recovery, more axons, and better remyelination compared to other groups, indicating its potential for nerve regeneration in peripheral nerve injuries [85].

Fibrin or synthetic poly(ethylene glycol) and fibrinogen/gelatin hydrogels with laser-ablated microchannels were utilized for sciatic nerve regeneration. The hydrogels were evaluated for shear modulus, biological composition, micropatterning for unidirectional growth, controlled degradation, and stiffness properties. Microchannel guidance patterns combined with matched material properties were essential for uniform tissue propagation during nerve regeneration, showing improved outcomes in subcritical nerve gap injuries in rats compared to unpatterned hydrogels [86].

Poly(2-hydroxyethyl methacrylate-co-methyl methacrylate) (PHEMA-MMA) porous tubes were developed for sciatic nerve repair. These tubes were characterized by their biocompatibility, pore structure, mechanical stability, and regenerative capacity. Axonal regeneration within PHEMA-MMA tubes was comparable to nerve grafts at 8 and 16 weeks, with a bimodal response observed in 16-week tubes, indicating their potential as an alternative to autografts for peripheral nerve repair [87].

Gelatin methacrylate (GelMA)/poly(2-ethyl-2-oxazoline) (PEtOx) hydrogel containing 4-aminopyridine (4-AP) was investigated for sciatic nerve injury. The hydrogel was assessed for porosity, swelling ratio, weight loss, blood compatibility, sustainable drug release, and the potassium channel-blocking properties of 4-AP. The GelMA/PEtOx+4-AP hydrogel showed enhanced regeneration in vivo, with improved sciatic functional index (SFI) and hot-plate latency results compared to the GelMA/PEtOx hydrogel and control group, indicating its potential for peripheral nerve recovery [88].

Anisotropic polyacrylamide (PAM) hydrogel micropatterns with aligned ridge/groove structures, biofunctionalized using YIGSR peptide, were created to guide Schwann cell behavior. These hydrogels were designed with anisotropic micropatterns for cell alignment and YIGSR peptide biofunctionalization and evaluated for swelling, mechanical properties, and stability. Biofunctionalized anisotropic hydrogel micropatterns effectively regulated the orientation growth of Schwann cells, upregulated BDNF and beta-actin expression, and maintained the normal secretion of neurotropic factors, providing a basis for artificial implants in nerve regeneration [89].

A pure silk fibroin hydrogel with an aligned microgrooved topographic structure was designed for peripheral nerve regeneration. The hydrogels exhibited excellent mechanical properties and biocompatibility, supporting Schwann cell survival and promoting axon growth and neurite sprouting, demonstrating great potential for peripheral nerve regeneration [34].

A photothermal-responsive cell-laden poly-N-isopropylacrylamide (PNIPAM) hydrogel containing dopamine hydrochloride-modified multi-walled carbon nanotubes (MWCNTs) was developed for nerve regeneration. The PNIPAM hydrogel with MWCNTs modified with 2 mg/mL dopamine showed the best photothermal responsiveness, good dispersibility, stability, hydrophilicity, and nontoxicity. Schwann cells grown in the 3D hydrogel showed better growth, released more nerve growth factors, and improved the capability of nerve grafts to repair peripheral nerve injuries [90].

A polyamidoamine (PAA) hydrogel scaffold shaped as a small tube was obtained by radical polymerization of a soluble functional oligomeric precursor and was investigated as a conduit for nerve regeneration in a rat sciatic nerve cut model. This study focused on the biocompatibility, biodegradability, low interfacial tension, tunable elasticity, conduit shape for nerve guidance, and non-inflammatory properties of the PAA hydrogel. The PAA hydrogel conduits facilitated nerve regeneration with good surgical outcomes, no inflammation or neuroma, and satisfactory functional recovery, indicating its potential as a novel material for peripheral nerve regeneration [91].

A polyacrylamide/silk fibroin/graphene oxide composite hydrogel was designed for Schwann cell culture and nerve regeneration. This composite hydrogel featured a three-dimensional network structure, hydrophilicity, wettability, porosity, and mechanical strength, which collectively provided a better growth environment for Schwann cells. Enhanced mechanical properties and improved cell growth environments suggest its potential for nerve regeneration and tissue engineering [92].

Magnesium-encapsulated injectable hydrogel combined with a polycaprolactone (PCL) conduit was investigated for nerve regeneration. The study focused on sustained Mg^2+^ delivery, the activation of the PI3K/Akt signaling pathway, and the role of PCL conduits in enhancing axon regeneration, remyelination, and functional recovery. The Mg^2+^-releasing hydrogel significantly improved axon regeneration, remyelination, and functional recovery in rats with peripheral nerve injuries, providing a promising strategy for nerve repair [93].

An in situ visible photo-crosslinkable protein-based bioadhesive hydrogel containing a functional neurotransmitter peptide was developed for sutureless neurorrhaphy. This system utilized a macrophage-polarizing visible-light-crosslinkable adhesive protein hydrogel for secure anastomosis and a reduced inflammatory response. The bioadhesive hydrogel provided effective sutureless anastomosis, induced M2 macrophage polarization, and significantly enhanced functional nerve regeneration compared to conventional sutures, offering a promising alternative for nerve regenerative medicine [94].

A hierarchically aligned fibrin nanofiber hydrogel (AFG) was prepared through electrospinning and molecular self-assembly for peripheral nerve regeneration. This hydrogel featured hierarchically aligned topography, low elasticity, and directional cell adhesion and migration, creating a supportive microenvironment for Schwann cells and axonal regrowth. The AFG supported Schwann cell cable formation and axonal regrowth, leading to improved motor functional recovery, with outcomes comparable to autologous nerve grafts and superior to hollow chitosan tubes and random fibrin nanofiber hydrogels [95].

Graphene foam/hydrogel scaffolds combined with adipose-derived stem cells (ADSCs) were investigated for peripheral nerve regeneration in a diabetic mouse model. These scaffolds provided mechanical strength, a porous network, electrical conductivity, and biocompatibility and regulated the Nrf2/HO-1, NF-κB, and PI3K/AKT/mTOR signaling pathways, reducing oxidative stress and inflammation. The ADSC-loaded GF/hydrogel scaffold significantly promoted the recovery of diabetic peripheral nerve injury and inhibited muscle atrophy, offering a novel and attractive therapeutic approach for diabetic peripheral nerve injury patients [96].

A graphene mesh-supported double-network (DN) hydrogel scaffold loaded with netrin-1 was engineered for nerve regeneration. Composed of natural alginate, gelatin-methacryloyl, and graphene mesh, this scaffold provided mechanical strength, biocompatibility, and electrical conductivity, with netrin-1 promoting axon pathfinding and neuronal migration. The netrin-1-loaded graphene mesh tube/DN hydrogel nerve scaffold significantly promoted peripheral nerve regeneration, demonstrating superiority to autografts [97].

Poly(2-hydroxyethyl methacrylate-co-methyl methacrylate) (PHEMA-MMA) coil-reinforced hydrogel tubes were synthesized and compared with autografts for nerve regeneration. Three designs—plain, corrugated, and coil-reinforced tubes—were evaluated for patency, regenerative capacity, electrophysiology, histomorphometry, nerve action potential (NAP) velocity, and muscle action potential (MAP) velocity. The coil-reinforced tubes demonstrated equivalent nerve regeneration to autografts, highlighting the importance of channel design and patency [98].

A gelatin methacryloyl (GelMA) hydrogel photocrosslinked under a blue-light source (405 nm) was studied for nerve repair and regeneration in mice with spinal cord injury. The hydrogel’s biocompatibility, photocrosslinking, exercise performance, axon length, syringomyelia reduction, and scar formation inhibition were assessed. The GelMA hydrogel promoted nerve regeneration, reduced syringomyelia, inhibited scar formation, and improved limb movement function in mice with SCI [99].

The various synthetic and composite hydrogels for neural regeneration exhibit several common features. Many studies incorporate conductive materials such as graphene oxide, polypyrrole, or carbon nanotubes to enhance electrical conductivity, which is beneficial for neural tissue engineering. Additionally, these hydrogels often include bioactive molecules like growth factors or utilize structural modifications such as aligned fibers or micropatterning to guide nerve regeneration. Most studies employ in vivo models, typically using rodents, to evaluate the hydrogels’ effectiveness in promoting nerve regeneration and functional recovery [85,86,87,88,91,93,94,96,99,100,101].

The comparative analysis of various synthetic and composite hydrogels for nerve regeneration suggests that the most effective compositions integrate conductive materials, bioactive molecules, and advanced structural designs. The inclusion of graphene oxide [82,85] and other conductive polymers has shown significant enhancements in nerve regeneration and functional recovery. Additionally, the use of structural modifications such as aligned fibers or micropatterning [84,89] provided notable improvements in Schwann cell proliferation and functional recovery. For instance, some hydrogels combine conductive materials with natural polymers like collagen or chitosan to enhance biocompatibility and support nerve regeneration [82,85], while others use synthetic polymers such as polyacrylamide or poly(2-hydroxyethyl methacrylate) to provide structural integrity and tunable mechanical properties [83,87].

Table 8 outlines various miscellaneous synthetic and composite hydrogels designed for neural regeneration, highlighting their physical, chemical, and biological properties. These hydrogels support nerve repair through features such as biocompatibility, mechanical strength, enhanced cell proliferation, and directional growth, making them suitable for treating peripheral nerve injuries and specific conditions like facial nerve and sciatic nerve injuries.

## 10. Specialized Hydrogels for Specific Applications

This section discusses the development and application of specialized hydrogels for nerve tissue engineering. It highlights various formulations, like GelMA, thermosensitive, agarose-methylcellulose blends, and chondroitin sulfate hydrogels. These hydrogels offer biocompatibility, biodegradability, and tunable mechanical properties, supporting nerve repair and regeneration through bioactive molecule incorporation and in vivo model testing.

A gelatin methacrylate (GelMA) hydrogel, a gelatin modified by methacrylamide, has been extensively reviewed for its applications in nerve tissue engineering. This hydrogel possesses adjustable mechanical properties, convenient processability, and excellent biocompatibility, making it ideal for 3D scaffold manufacturing. GelMA-based hydrogels have shown promising results in promoting nerve repair and regeneration, supporting the use of these materials as scaffolds for seed cells and active factors in nerve tissue engineering [102].

Further advancements in GelMA hydrogels include their modification with methacrylic anhydride and loading with vascular endothelial growth factor (VEGF). This formulation exhibits good physical and chemical properties and biocompatibility and supports cell adhesion and proliferation. The controlled release of VEGF from the hydrogel promoted nerve regeneration, functional recovery, and vascularization in a rat sciatic nerve crush injury model, highlighting the potential of this approach for effective nerve repair [103].

A thermosensitive hydrogel (PALDE) carrying extracellular vesicles (EVs) from adipose-derived stem cells was studied for peripheral nerve regeneration after microsurgical repair. This hydrogel promotes Schwann cell migration and proliferation, as well as axon outgrowth. It solidifies rapidly and sustains a high EV concentration around the repaired nerve, enhancing nerve conduction efficacy and muscle contraction force in a rat sciatic nerve repair model. The EV-loaded thermosensitive hydrogel showed significant potential in promoting peripheral nerve regeneration [104].

Agarose and methylcellulose hydrogel blends have been created for nerve regeneration applications. These thermoreversible hydrogels combine methylcellulose with agarose to create injectable materials at room temperature that solidify at physiological temperatures (37 °C). The blends solidify faster and have a higher elastic modulus compared to base methylcellulose. These properties support the morphology of dissociated dorsal root ganglion neurons and hold the potential for delivering therapeutics and maintaining scaffolds in place during nerve regeneration [105].

Chondroitin sulfate hydrogel has been designed as a scaffold for regenerating root neurons and delivering neurotrophic signals. This hydrogel shows a strong affinity with common neurotrophins and provides a better scaffold for neurite outgrowth compared to hyaluronic acid. The chondroitin sulfate hydrogel supported more robust growth of cultured ganglia, indicating its superior potential as a scaffold for neurite outgrowth and nerve root regeneration [106].

Specialized hydrogels for specific applications in nerve regeneration share several common features. These hydrogels are designed to be biocompatible, biodegradable, and possess tunable mechanical properties, making them suitable for various neural tissue engineering applications. They often incorporate bioactive molecules, such as growth factors, extracellular vesicles, or neurotrophic signals, to enhance their regenerative capabilities. Additionally, these hydrogels are typically evaluated using in vivo models to assess their effectiveness in promoting nerve repair and functional recovery [27,103,104,105,106].

The comparative analysis of specialized hydrogels for specific applications in nerve tissue engineering suggests that the most effective compositions integrate bioactive molecules, exhibit tunable mechanical properties, and ensure biocompatibility and biodegradability. GelMA hydrogels, particularly when modified with methacrylic anhydride and loaded with VEGF, have shown significant enhancements in nerve repair, functional recovery, and vascularization [103]. Thermosensitive hydrogels carrying extracellular vesicles from adipose-derived stem cells also demonstrated substantial improvements in Schwann cell migration, axon outgrowth, and muscle function, making them highly effective for peripheral nerve regeneration [104]. Additionally, agarose and methylcellulose hydrogel blends offer advantageous thermoreversible properties and structural stability at physiological temperatures, supporting nerve regeneration and therapeutic delivery [105]. Chondroitin sulfate hydrogels excel in delivering neurotrophic signals and promoting neurite outgrowth, providing a superior scaffold for nerve root regeneration [106].

Table 9 highlights specialized hydrogels designed for specific neural regeneration applications, detailing their physical, chemical, and biological properties. These hydrogels support nerve repair by offering features like adjustable mechanical properties, biocompatibility, rapid solidification, and the ability to sustain the release of growth factors and extracellular vesicles, making them suitable for treating various nerve injuries, including peripheral nerve crush injuries and central nervous system injuries.

## 11. Factors Affecting the Selection of Hydrogels for Neural Regeneration

The selection of hydrogels for neural regeneration is influenced by several key factors that help tailor these materials to address specific neural injuries and regeneration challenges. These factors include the type of nerve injury, hydrogel properties, additive components, delivery methods, and biocompatibility and safety.

Different neural injuries require hydrogels with specific properties. For instance, spinal cord injuries often benefit from hydrogels that support electrical signal transmission and promote axonal growth, such as conductive hydrogels [13,80]. In contrast, peripheral nerve injuries prioritize mechanical properties, biocompatibility, and support for Schwann cell activity and myelination [8,28,41,82]. This differentiation ensures that the hydrogel properties are aligned with the unique needs of the injury type, optimizing the chances for effective regeneration.

The physical properties of hydrogels, such as porosity, biodegradability, and mechanical strength, are critical for creating an environment conducive to nerve regeneration. Tailoring these properties can enhance cell proliferation and tissue integration [39,41,51,75]. For example, aligned nanofibers in the hydrogel structure can efficiently guide axonal growth [34,39], while appropriate biodegradability ensures that the hydrogel degrades at a suitable rate to support tissue regeneration [22]. Mechanical strength is also essential for providing structural support, especially in large nerve defects [31,69].

Incorporating growth factors, drugs, conductive materials, and bioactive molecules into hydrogels significantly enhances their regenerative capabilities. Growth factors like NGF and BDNF improve neuronal survival and promote tissue repair [41,47,68]. Conductive materials such as graphene oxide and polypyrrole enhance electrical conductivity, supporting neural cell functions and regeneration [51,52]. Additionally, bioactive molecules, including neurotrophic peptides, antioxidants, and anti-inflammatory agents, support cell proliferation, reduce inflammation, and enhance neuroprotection [14,15,58]. The choice of additives, such as 4-methylcatechol, berberine, and hesperidin, can also enhance nerve regeneration through various mechanisms, including neuroprotection and anti-inflammatory effects [22,23,32,45].

Effective delivery methods and sustained release profiles are crucial for the therapeutic success of hydrogels. Injectable hydrogels offer minimally invasive delivery and better tissue integration, which is particularly advantageous for spinal cord injuries [13,14,48,68]. Preformed hydrogel conduits are often preferred for bridging larger nerve gaps, providing essential structural support [9,39]. Controlled release systems ensure sustained therapeutic effects by enabling the prolonged release of growth factors, drugs, or cells, maintaining their effectiveness over extended periods [27,28,31,73]. For example, alginate/gum Arabic hydrogels with SiNGF enable the prolonged release of nerve growth factors, enhancing tissue regeneration [24].

Ensuring the biocompatibility and safety of hydrogels is paramount. Rigorous in vitro and in vivo testing confirms the cytocompatibility and hemocompatibility of the hydrogels, ensuring they are nontoxic and do not elicit adverse immune responses [8,32,41,51]. This testing is critical for evaluating any potential long-term side effects and ensuring that the hydrogels support cell viability and proliferation without causing harm. Studies often assess these factors through various in vitro and in vivo tests [8,14,15,23,26,40,41,43,45,46,47,48,56,58,60].

By carefully considering these factors—the type of nerve injury, hydrogel properties, additive components, delivery methods, and biocompatibility—researchers can optimize hydrogels to meet the specific needs of neural regeneration. This tailored approach enhances the effectiveness of hydrogels in promoting nerve repair and regeneration, improving outcomes for patients with neural injuries.

## 12. Testing Hydrogels for Neural Regeneration and Repair

Hydrogels have emerged as promising materials for neural regeneration and repair due to their biocompatibility, tunable mechanical properties, and ability to mimic the natural extracellular matrix. Comprehensive testing is crucial for evaluating their efficacy and safety. This includes in vitro and in vivo tests, providing critical insights into different aspects of hydrogel performance.

### 12.1. In Vitro Tests

In vitro tests focus on the morphological, mechanical, and biological properties of hydrogels. Morphological analysis using scanning electron microscopy (SEM) is essential to ensuring the appropriate pore size and interconnectivity for cell growth and nutrient transport [10,56]. The hemocompatibility and cytocompatibility of hydrogels are evaluated through hemocompatibility tests and cytocompatibility assays, such as MTT and XTT, to confirm that the hydrogel does not induce adverse cellular or blood responses [8,32].

Mechanical and degradation properties are also critical. Mechanical properties such as the tensile strength, compressive modulus, and Young’s modulus are measured to ensure that the hydrogel can withstand physiological conditions without degrading prematurely [34,55]. Additionally, degradation rate tests measure the rate at which the hydrogel degrades over time, which is crucial for ensuring that the material supports neural regeneration for an adequate period [22,55].

Cell viability and proliferation are assessed using various assays. Viability assays like MTT and resazurin analyses help determine the hydrogel’s ability to support cell survival and proliferation, while LIVE/DEAD staining provides additional insights into cell viability [29,32]. Neurite outgrowth assays are used to evaluate neurite extension and the bioactivity of neural growth factors, confirming the hydrogel’s capability to promote neural differentiation and network formation [46,84].

The electrophysiological and conductivity properties of hydrogels are evaluated to ensure their suitability for neural applications. Electrophysiological recordings, such as compound muscle action potential (CMAP) latency and nerve conduction velocity (NCV), assess the hydrogel’s ability to support and transmit electrical signals [61,67]. Conductivity measurements are also conducted to ensure that the hydrogel can support neural signal transmission [96,97].

### 12.2. In Vivo Tests

In vivo tests provide insights into the functional recovery and integration of hydrogels in living organisms. Functional recovery is often assessed using the sciatic functional index (SFI), which measures motor function recovery in animal models [9,42]. Behavioral tests, such as walking-footprint analysis and hot-plate latency tests, evaluate sensory and motor function recovery post-implantation [8,45].

Histological and biochemical analyses are conducted to observe tissue integration, axonal regeneration, and inflammatory responses. Techniques such as immunohistochemistry and Masson’s trichrome staining are used for detailed histological assessments [14,40,48]. Additionally, biochemical markers like cytokine levels and gene-expression analysis are measured to study the inflammatory and regenerative responses induced by the hydrogel [14,15,48].

Muscle mass and function, such as gastrocnemius muscle mass measurements, serve as indicators of successful nerve regeneration and functional recovery [9,26].

## 13. Collective Outcomes

Hydrogels have demonstrated significant advancements in nerve regeneration through enhanced biocompatibility, structural integrity, and functional recovery. Studies report high biocompatibility, minimal toxicity, and positive biological interactions, such as with thiolated chitosan/taurine hydrogels and chitosan conduits filled with simvastatin/Pluronic F-127 hydrogel, which improved sciatic nerve regeneration and increased neurotrophic factors [8,9]. Hydrogels like LiCl-loaded hyaluronic acid and HA-PVA-Hep promote Schwann cell proliferation and axonal growth due to their high biocompatibility and stability [42,58,79].

Hydrogels possess favorable structural and mechanical properties, such as matching the Young’s modulus of nerve tissues and supporting cell regrowth, exemplified by a chitosan/beta-glycerophosphate/salt hydrogel [55]. Tailored structural properties, like longitudinally oriented microchannels in decellularized nerve matrix hydrogels, improve neurite extension and Schwann cell migration [73].

Functional recovery and nerve regeneration have significantly improved with various hydrogels. Examples include AFG-prefilled chitosan tubes and a Schwann cell-encapsulated chitosan/collagen hydrogel nerve conduit (CCN) showing superior motor function recovery and axonal regrowth [12,39] and RADA16-I and RADA16-Mix hydrogels promoting better peripheral nerve regeneration in rats [61,62]. The self-healing electroconductive HASPy hydrogel and aldehyde-based hyaluronic acid with polyaniline (PANI) on a carboxymethyl chitosan (CMCS) ACCP3 hydrogel have also enhanced functional recovery and nerve regeneration [35,36].

Incorporating advanced materials like BP nanosheets, peptide amphiphile nanofibers, and graphene oxide composites significantly improves hydrogel properties, enhancing conductivity, mechanical stability, and neuroprotective effects [13,43,51,78]. Conductive hydrogel scaffolds with wireless electrical stimulation promote remyelination and neural stem cell differentiation [80], while composite hydrogels with materials like MWCNTs and fibroblast exosomes improve mechanical properties and cell growth environments [54,90].

Hydrogels with controlled release mechanisms and antimicrobial properties show promising results in promoting nerve regeneration while preventing infections. Examples include an AG-Car/SiNGF hydrogel for sustained NGF release and reduced inflammation [24] and a GelMA/PEtOx hydrogel with 4-AP for controlled drug release and enhanced functional recovery [88]. A Mg^2+^-releasing hydrogel combined with a 3D-engineered PCL conduit demonstrates the potential for controlled ion release in neural repair [93].

Innovative structural designs, such as microchannel guidance patterns and hierarchically aligned fibrin nanofiber hydrogels, improve tissue propagation and directional cell migration, further enhancing nerve regeneration and functional recovery [86,95].

## 14. Collective Limitations

Hydrogels face significant challenges in nerve regeneration applications. There is notable variability in outcomes, with some hydrogels like the CS/GP hydrogel showing positive in vitro results but inconsistent results in vivo [40]. Biodegradation rates and structural stability often do not match the required nerve regeneration timelines, with some hydrogels degrading too quickly to provide sustained support [8,45]. The complexity and scalability of producing advanced hydrogels, particularly those with integrated nanomaterials, pose significant barriers to their widespread clinical use. Additionally, ensuring compatibility with host tissue while minimizing immune responses remains critical, as some hydrogels can cause inflammatory reactions or fail to integrate properly. Finally, matching the mechanical properties and degradation rates of hydrogels to the needs of neural tissue is challenging, with many hydrogels failing to provide adequate support for regenerating nerves [52].

## 15. Future Directions

Future research in hydrogel-based nerve regeneration should focus on optimizing physical and chemical properties to better mimic natural nerve tissue by fine-tuning pore sizes, degradation rates, and mechanical properties [13,24,27,73]. Developing multifunctional hydrogels that combine biocompatibility, the controlled release of growth factors, and antimicrobial properties while integrating advanced materials like conductive nanofibers and bioactive nanoparticles can enhance therapeutic outcomes [13,24]. Bridging the gap between in vitro success and in vivo performance is crucial, necessitating rigorous in vivo testing and long-term studies to validate efficacy and safety while addressing immune responses and ensuring stable integration with host tissues [40,41]. Personalized and adaptive hydrogels that are tailored to individual patient needs and dynamically responsive to the healing environment can significantly improve outcomes. Continued interdisciplinary collaboration among bioengineering, materials science, and neuroscience experts is essential to foster innovation and accelerate the development of next-generation hydrogels for neural regeneration.

## 16. Conclusions

Hydrogels such as chitosan, alginate, collagen, hyaluronic acid, and peptide amphiphiles exhibit high biocompatibility and biodegradability, which are essential for nerve repair. These materials support cell adhesion and proliferation, mimicking the extracellular matrix (ECM) to promote nerve regeneration. Various formulations have improved functional recovery, axonal regrowth, and myelination. Advances include pure silk fibroin hydrogels, gelatin membranes, granular hydrogels, nanofiber scaffolds, and composite hydrogels with graphene oxide. Innovative techniques like 3D bioprinting enhance the scaffold architecture. However, clinical translation faces challenges, such as inconsistent efficacy, complex fabrication, and limited long-term studies. Future research should optimize hydrogel properties, develop multifunctional hydrogels, integrate stem cell therapy, and conduct extensive clinical trials for neural regeneration.

## Figures and Tables

**Table 1:** Chitosan-based hydrogels in neural regeneration.

Hydrogel Structure	Hydrogel Physical, Chemical, and Biological Characteristics	Neural Disease/Disorder Targeted	Ref
Thiolated chitosan hydrogel containing taurine	Interconnected pores, biocompatibility, biodegradability, sustained release of taurine	Peripheral nerve gap	[8]
Chitosan conduits filled with simvastatin/Pluronic F-127 hydrogel	Scaffold support, increased neurotrophic factors	Peripheral nerve gap	[9]
Carboxymethyl chitosan hydrogel (radiation-induced crosslinking)	Biocompatibility, antimicrobial properties, hydrophilic properties, scaffold support	Peripheral nerve regeneration	[10]
Injectable black phosphorus nanosheets and lipoic acid-modified chitosan hydrogel loaded with tazarotene (LAMC/BP@TA)	Conductivity, angiogenic potential, neurogenic potential	Spinal cord injury	[13]
Substance P-conjugated chitosan hydrochloride hydrogel (CSCl-SP)	Stability of SP, angiogenesis, extracellular matrix deposition, nerve regeneration	Full-thickness skin defects	[21]
Chitosan tubes prefilled with aligned fibrin nanofiber hydrogel (AFG)	Mimics nerve ECM, supports adhesion and proliferation of Schwann cells, promotes axonal growth	Facial nerve injury	[39]
Chitosan/beta-glycerophosphate/salt hydrogel with aligned conductive nanofibers (polycaprolactone/gelatin/SWCNTs)	Conductivity, mechanical properties matching neural tissue, interconnected structure	Nerve tissue regeneration	[55]

**Table 2:** Alginate-based hydrogels in neural regeneration.

Hydrogel Structure	Hydrogel Physical, Chemical, and Biological Characteristics	Neural Disease/Disorder Targeted	Ref
Alginate/chitosan hydrogel containing 4-methylcatechol	Pore size suitable for cell proliferation, biodegradability, hemocompatibility	Peripheral nerve regeneration	[22]
Chitosan/alginate hydrogel with berberine and naringin nanoparticles	Swelling, porous, weight loss, mechanical strength, controlled release, biocompatibility	Peripheral nerve regeneration	[23]
Alginate/gum arabic hydrogel with immobilized NGF and carnosine	Sustained NGF release, reduced inflammation, increased angiogenesis, enhanced re-epithelialization	Diabetic foot ulcers	[24]
Alginate hydrogel scaffold with melatonin	Controlled release, ECM-like microenvironment, antioxidant properties, reduced inflammation, improved angiogenesis	Peripheral nerve injury	[28]
Alginate/magnetic short nanofiber composite hydrogel encapsulating olfactory ecto-mesenchymal stem cells (OE-MSCs)	Mimics ECM, enhances cell viability and proliferation, promotes neural-like differentiation	Nerve regeneration	[29]
Alginate/chitosan hydrogel with berberine	Pore size, controlled release, hemocompatibility, cytocompatibility, biocompatibility, promotion of cell proliferation	Peripheral nerve regeneration	[32]
Alginate/chitosan hydrogel with hesperidin	Interconnected porosity, biodegradability, blood compatibility, antibacterial properties	Peripheral nerve regeneration	[45]

**Table 3:** Collagen-based hydrogels in neural regeneration.

Hydrogel Structure	Hydrogel Physical, Chemical, and Biological Characteristics	Neural Disease/Disorder Targeted	Ref
Chitosan/collagen hydrogel hybrid nerve guidance conduit containing Schwann cells	Mechanical strength, bioabsorbable, biocompatible; supports axonal extension	Peripheral nerve regeneration	[11]
Chitosan/collagen hydrogel nerve conduit encapsulating Schwann cells	Synergistic effect on axonal regrowth and remyelination, biocompatibility, mechanical support	Peripheral nerve regeneration	[12]
Collagen type I hydrogel containing chitosan nanoparticles loaded with insulin	Increases Schwann cell proliferation and muscle action potential, promotes nerve regeneration	Peripheral nerve regeneration	[25]
Collagen type I hydrogel containing naringin	Promotes Schwann cell proliferation, porous structure, swelling behavior, cyto/hemocompatibility, biodegradability	Peripheral nerve regeneration	[30]
Hydroxyapatite nanoparticle-containing collagen type I hydrogel	Enhances Schwann cell proliferation, biocompatibility	Peripheral nerve regeneration	[33]
Nanofiber neural guidance channels with collagen hydrogel and acetyl L-carnitine (ALC)	Supports cell attachment and tensile strength, has favorable effects on metabolic activity	Peripheral nerve injury	[56]
Porous PLCL membrane with 3D-printed collagen hydrogel	Elasticity, aligned collagen fibers, longitudinal orientation, nanoporous structure; promotes axonal regeneration and remyelination	Peripheral nerve regeneration	[57]

**Table 4:** Hyaluronate-based hydrogels in neural regeneration.

Hydrogel Structure	Hydrogel Physical, Chemical, and Biological Characteristics	Neural Disease/Disorder Targeted	Ref
Hyaluronan/methylcellulose hydrogel with anti-inflammatory peptide and brain-derived neurotrophic factor (BDNF)	Minimally swelling, sustained release; reduces inflammation, promotes neuronal survival	Spinal cord injury	[14]
2,2,6,6-Tetramethylpiperidinyloxy (TEMPO)-functionalized hyaluronic acid hydrogel	Antioxidant effect; promotes tissue reconnection, protects bladder tissue	Spinal cord transection	[15]
Chitosan/hyaluronic acid hydrogel for nerve growth factor (NGF) sustained release	Rapid gelation, interconnected channels, controllable pore diameter, suitable swelling and degradation behavior	Peripheral nerve regeneration	[41]
Lithium-loaded hyaluronic acid hydrogel	Neuroprotective, anti-inflammatory; promotes nerve regeneration	Peripheral nerve injury	[42]
Matrix metalloproteinase-sensitive, hyaluronic acid-based biomimetic hydrogel containing BDNF	Promotes stem cell differentiation, contains IKVAV peptide and BDNF, supports neural differentiation	Spinal cord injury	[48]
Hyaluronic acid/phenylboronic acid/poly (vinyl alcohol)/heparin hydrogel loaded with glial cell-derived neurotrophic factor (GDNF)	Injectable, antioxidative, anti-inflammatory, biocompatible, sustained release; supports nerve regeneration	Peripheral nerve injury	[58]
Hyaluronic acid granular hydrogel nerve guidance conduit	Simulates extracellular matrix, promotes axonal extension.	Peripheral nerve injury	[59]

**Table 5:** Peptide-based hydrogels in neural regeneration.

Hydrogel Structure	Hydrogel Physical, Chemical, and Biological Characteristics	Neural Disease/Disorder Targeted	Ref
Peptide amphiphile nanofiber hydrogel with sonic hedgehog protein (SHH)	Supports nerve regeneration, prevents neuronal degeneration, maintains glial signaling	Cavernous nerve injury (prevents erectile dysfunction)	[43]
Neurotrophic peptide-functionalized self-assembling peptide nanofiber hydrogel (RAD/RGI)	Promotes cell adhesion, proliferation, neuronal differentiation, axonal regeneration	Sciatic nerve regeneration	[44]
Peptide amphiphile (PA)-based hydrogel consisting of C16GSH	Biocompatibility; supports Schwann cell proliferation and migration, provides angiogenesis support	Peripheral nerve injury	[60]
Peptide RADA16-I nanofiber scaffold hydrogel	Promotes faster healing	Peripheral nerve injury	[61]
Self-assembling peptide nanofiber hydrogel (RADA 16-Mix)	Enhanced axonal regeneration, Schwann cell migration, better functional recovery	Peripheral nerve injury	[62]
RADA16-I self-assembling peptide hydrogel	Supports neurite outgrowth and functional synapse formation, increases myelinated nerve areas	Recurrent laryngeal nerve regeneration	[63]
BD PuraMatrix peptide hydrogel seeded with Schwann cells	Supports Schwann cell survival and proliferation, increases regeneration distance	Peripheral nerve injury	[64]
Multidomain peptide (MDP) hydrogels in PCL conduits	Anionic MDP: may improve functional recovery; cationic MDP: poor functional recovery, higher muscle weight retention, better myelination	Transected peripheral nerve injury	[65]
Silk fibroin peptide (SF16) hydrogel	Biocompatible, impressive mechanical and degradative properties; enhances nerve regeneration, maintains cell viability, promotes axonal growth	Peripheral nerve injury	[66]
Human hair keratin hydrogel scaffold	Enhances motor recovery; larger nerve area, higher myofiber density	Peripheral nerve injury	[67]

**Table 6:** Hydrogels loaded with specific growth factors and cells in nerve regeneration.

Hydrogel Structure	Hydrogel Physical, Chemical, and Biological Characteristics	Neural Disease/Disorder Targeted	Ref
Poly(D, l-lactic acid) (PDLLA)/β-tricalcium phosphate (β-TCP) nerve conduits filled by injectable chitosan (CS)/hyaluronic acid (HA) hydrogels with NGF	Sustained NGF release; supports neuronal cell adhesion, spreading, and differentiation	Peripheral nerve injury	[26]
Hydrogel-enriched chitosan conduits with Schwann cells containing glial cell line-derived neurotrophic factor (GDNF) and fibroblast growth factor 2 (FGF-2)	Biocompatible; supports axonal outgrowth, SC viability, cellular neurotrophic factor delivery system	Peripheral nerve injury	[46]
PHEMA-MMA tubes with collagen gel and growth factors (neurotropin-3, brain-derived neurotrophic factor (BDNF), and acidic fibroblast growth factor (FGF-1))	Supports nerve regeneration; biostable, biocompatible, improved regeneration with growth factors	Peripheral nerve injury	[47]
Chitosan/beta-glycerophosphate-NGF hydrogel	Continuous NGF release, improved nerve recovery, thicker myelin sheaths	Facial nerve injury	[49]
Mesenchymal stem cell-laden fibrin hydrogel microfibers	Mimic nerve tissue, control stem cell behavior, improve motor function, and promote integration with the host tissue	Spinal cord injury	[50]
Injectable thermosensitive CS/beta-GP/HEC hydrogel containing nerve growth factor (NGF)-overexpressing hADSCs	Increased cell proliferation, smaller cavity size, better locomotor functions	Spinal cord injury	[68]
Erythropoietin (EPO)-loaded multifunctional hydrogel with adipose-derived stem cells (ADSCs)	Adhesive, high mechanical strength, sustained release; enhances cell retention, supports nerve regeneration, reduces astrocyte development	Neurogenic erectile dysfunction	[69]
Poloxamer hydrogel with adipose-derived stem cells (ASCs)	Promotes axonal regrowth, supports muscle reinnervation; beneficial immunomodulation	Peripheral nerve injury	[70]
Heparin/Poloxamer thermosensitive hydrogel loaded with bFGF and NGF	Provides supportive environment to restore structure, enables sustained release of growth factors, enhances axonal regeneration, supports Schwann cell proliferation	Peripheral nerve injury in diabetic rats	[71]
Decellularized porcine nerve-derived hydrogel (PNM)	Maintains nerve-specific matrix components and growth factors, improves electrophysiologic response and axon counts	Nerve gap injury	[72]
Decellularized nerve matrix hydrogel derived from porcine sciatic nerve with microchannels (A-pDNM-G)	Longitudinally oriented microchannels, promotion of neurite extension and Schwann cell migration, controlled release of neurotrophic factors	Peripheral nerve injury	[73]
Fibrin hydrogel with tacrolimus and mesenchymal stem cells (MSC)	Sustained and localized drug release; supports MSC viability, enhances peripheral nerve regeneration	Peripheral nerve injury	[74]
Gelatin hydrogel with olfactory stem cells (OSC)	Biodegradable; aids cell survival during transplantation, promotes axonal regeneration, increases number of regenerated nerve fibers	Facial nerve injury	[75]

**Table 7:** Conductive hydrogels in neural regeneration.

Hydrogel Structure	Hydrogel Physical, Chemical, and Biological Characteristics	Neural Disease/Disorder Targeted	Ref
HA-modified polypyrrole self-healing hydrogel (HASPy)	Injectability, biodegradability, biocompatibility; targets IL-17 signaling pathway, promotes remyelination	Peripheral nerve injury	[35]
Injectable self-healing hydrogel with polyaniline (PANI) on carboxymethyl chitosan (CMCS) and aldehyde-based hyaluronic acid (ALHA) (ALHA/CMCS/CP)	Biocompatibility, elastic modulus, good electrical conductivity; supports Schwann cell proliferation and migration, reduces tissue resistivity	Peripheral nerve injury	[36]
Conductive immunomodulatory hydrogel bandage composed of extracellular matrix, oxidized polysaccharides, poly(3,4-ethylenedioxythiophene)/poly(styrenesulfonate) (PEDOT:PSS) and loaded with Indole-3-propionic acid (IPA)	Adhesive, conductive, self-assembled; modulates immune response, supports axonal growth, prevents muscle atrophy	Peripheral nerve regeneration	[37]
Chitosan/oxidized hydroxyethyl cellulose/reduced graphene oxide/asiaticoside liposome hydrogel	Conductive, nontoxic; promotes adhesion and proliferation of nerve cells, inhibits scar formation, enhances electrical stimulation	Peripheral nerve regeneration	[51]
Plasticine-like conductive hydrogel composed of gelatin, polypyrrole, and tannic acid	Shape-persistent, self-healing, enhanced axonal regeneration and remyelination	Peripheral nerve injury	[52]
Reduced graphene oxide/gelatin-methacrylate hydrogel (r(GO/GelMA))	Conductive, flexible, mechanically stable, and permeable, which makes it suitable for use as NGCs; promotes neuritogenesis	Peripheral nerve regeneration	[53]
Silk fibroin hydrogel with graphene oxide and fibroblast exosome	Enhances electron transmission, modulates cellular behaviors, promotes vascular regeneration	Peripheral nerve regeneration	[54]
Aniline pentamer-crosslinked chitosan (CS-AP) hydrogel	Electroactive, better degradation; promotes capillary formation, supports nerve cell cytocompatibility, enhances nerve repair and muscle reinnervation	Peripheral nerve injury	[76]
Sodium alginate/carboxymethyl chitosan hydrogel with polypyrrole (SA/CMCS/PPy)	Adjustable conductivity, biocompatibility; supports cell growth with electrical stimulation	Peripheral nerve regeneration	[77]
Graphene oxide/oligo(polyethylene glycol fumarate) hydrogel (GO-OPF)	Electrically conductive, modulated properties; supports nerve cell adhesion and proliferation, enhances neurite extension	Nerve regeneration	[78]
Zwitterionic conductive hydrogel-based NGC	Mechanical stability, electrical conductivity, good cytocompatibility; promotes Schwann cell proliferation and neurite length	Peripheral nerve regeneration	[79]
Capacitive-coupling-responsive hydrogel scaffold	Wireless in situ electrical stimulation, biodegradable; promotes remyelination, accelerates axon regeneration,	Spinal cord injury	[80]
Black phosphorus (BP) hydrogel NGCs loaded with neuregulin 1 (Nrg1)	Good flexibility; promotes Schwann cell proliferation, supports neuron-branch elongation	Peripheral nerve regeneration	[81]

**Table 8:** Miscellaneous synthetic and composite hydrogels in neural regeneration.

Hydrogel Structure	Hydrogel Physical, Chemical, and Biological Characteristics	Neural Disease/Disorder Targeted	Ref
Pure silk fibroin hydrogel with aligned microgrooved structure	High mechanical strength, biocompatibility; supports aligned Schwann cell growth	Peripheral nerve regeneration	[34]
Ammonia-functionalized graphene oxide and frankincense-embedded hydrogel	Synergistic effect on axon regrowth; increases regenerating axons and myelin thickness	Facial nerve regeneration	[82]
Polyacrylamide/chitosan composite hydrogel	Elasticity and topographical guidance; promotes dorsal root ganglion neurite growth and better-oriented status	Peripheral nerve regeneration	[83]
Collagen/terpolymer hydrogel-filament scaffolds	Enhanced neurite extension, directional growth along fibers, higher levels of Na channels	Peripheral nerve regeneration	[84]
Polyacrylonitrile (PAN) conduit with fibrin hydrogel and graphene quantum dots (GQDs)	Promotes Schwann cell infiltration, improves sensorial recovery, enhances axon numbers and remyelination	Sciatic nerve injury	[85]
Photo-patterned hydrogels with microchannels (fibrin, PEG/fibrinogen, gelatin)	Provides contact guidance, controlled environmental stimuli, uniform tissue propagation, unidirectional growth	Sciatic nerve injury	[86]
Poly(2-hydroxyethyl methacrylate-co-methyl methacrylate) (PHEMA-MMA) hydrogel	Supports nerve regeneration comparable to autografts, promotes axonal regeneration	Sciatic nerve injury	[87]
GelMA/PEtOx hydrogel with 4-Aminopyridine (4-AP)	High porosity, sustainable drug release, good blood compatibility, enhances neuromuscular function	Sciatic nerve injury	[88]
Biofunctionalized anisotropic PAM hydrogel with YIGSR peptide	Adhesive; directionally induces cell alignment, accelerates cell migration, upregulates BDNF and beta-actin expression	Peripheral nerve regeneration	[89]
Poly-N-isopropylacrylamide (PNIPAM) hydrogel with dopamine-modified MWCNTs	Photothermal responsiveness, porous, good hydrophilicity; supports Schwann cell growth and nerve growth factor release	Peripheral nerve injury	[90]
Polyamidoamine (PAA) hydrogel	Biocompatible, biodegradable, tunable elasticity; facilitates nerve regeneration, with no inflammation or neuroma	Sciatic nerve injury	[91]
Polyacrylamide/silk fibroin/graphene oxide composite hydrogel	Excellent 3D network structure, hydrophilicity, wettability, and porosity, enhanced mechanical strength; supports Schwann cell growth	Peripheral nerve regeneration	[92]
Bisphosphonate-based injectable hydrogel with magnesium (Mg)	Sustained Mg^2+^ delivery; promotes neurite outgrowth via PI3K/Akt pathway, enhances axon regeneration and remyelination	Peripheral nerve injury	[93]
Macrophage-polarizing in situ visible-light-crosslinkable adhesive protein hydrogel containing functional neurotransmitter peptide	In situ crosslinking, macrophage polarization, effective tissue remodeling, sutureless anastomosis	Peripheral nerve injury (PNI)	[94]
Three-dimensional hierarchically aligned fibrin nanofiber hydrogel	Hierarchically aligned topography, low elasticity; mimics nerve ECM, promotes rapid directional cell adhesion	Peripheral nerve injury (PNI)	[95]
Graphene foam/hydrogel-based scaffold loaded with adipose-derived stem cells	Excellent mechanical strength, suitable porous network, superior electrical conductivity, good biocompatibility	Diabetic peripheral nerve injury (DPNI)	[96]
Graphene mesh-supported double-network (DN) hydrogel scaffold using natural hydrogel conduit (alginate and gelatin-methacryloyl) loaded with netrin-1	Good flexibility, Young’s modulus of 725.8 ± 46.52 kPa, electrical conductivity of 6.8 ± 0.85 S/m, UV crosslinking, biocompatible	Peripheral nerve injury	[97]
Poly(2-hydroxyethyl methacrylate-co-methyl methacrylate) (PHEMA-MMA) coil-reinforced hydrogel tubes	Patency, structural reinforcement, comparable mechanical properties to autografts	Peripheral nerve injury	[98]
Gelatin methacryloyl (GelMA) hydrogel	Photocrosslinkable, 10% (*w*/*v*), initiated under blue light (405 nm), good mechanical properties	Spinal cord injury	[99]
3D-printed polymeric hydrogels	Customizable structures, improved nerve reparation through bioprinting	Nerve cell regeneration	[100]

**Table 9:** Hydrogels designed for special neural regeneration applications.

Hydrogel Structure	Hydrogel Physical, Chemical, and Biological Characteristics	Neural Disease/Disorder Targeted	Ref
Gelatin methacrylate (GelMA) hydrogel	Adjustable mechanical properties, excellent biocompatibility, processability	Nerve injury	[102]
Gelatin modified by methacrylic anhydride hydrogel loaded with vascular endothelial growth factor	Good physical and chemical properties, biocompatibility; supports adhesion and proliferation of RSC96 cells	Peripheral nerve crush injury	[103]
Thermosensitive hydrogel (PALDE) loaded with extracellular vesicles from adipose-derived stem cells	Rapid solidification, sustained high concentration of extracellular vesicles (EVs) around repaired nerve	Peripheral nerve injury	[104]
Agarose and methylcellulose hydrogel blends	Rapid solidification at physiological temperatures, thermoreversible, delivering therapeutics, holding scaffolds	Nerve injury within CNS	[105]
Chondroitin sulfate hydrogel loaded with nerve growth factor (NGF)	Dissociation constant in micromolar range with neurotrophins, better scaffold for neurite outgrowth than hyaluronic acid	Brachial plexus injury	[106]

## Data Availability

No new data were created or analyzed in this study. Data sharing is not applicable to this article.

## Abbreviations

4-MC	4-Methylcatechol
ADSCs	Adipose-derived stem cells
AFG	Aligned fibrin nanofiber hydrogels
ASC	Adipose-derived stem cells
BDNF	Brain-derived neurotrophic factor
bFGF	Basic fibroblast growth factor
BMMSCs	Bone marrow-derived mesenchymal stem cells
BP@TA	Tannic acid-modified black phosphorus nanosheets
CMAP	Compound motor action potential
CMCS	Carboxymethyl chitosan
CNS	Central nervous system
CS/GP	Chitosan/glycerol-beta-phosphate disodium salt hydrogel
CSCl-SP	Substance P-conjugated chitosan hydrochloride hydrogel
DN	Double network
ECM	Extracellular matrix
ES	Electrical stimulation
FGF	Fibroblast growth factor
GDNF	Glial cell-derived neurotrophic factor
GelMA	Gelatin-methacrylate
GOA	Graphene oxide acrylate
HA	Hyaluronic acid
hADSCs	Human adipose-derived mesenchymal stem cells
HASPy	Self-healing electroconductive hydrogel
MDP	Multidomain peptide
MSCs	Mesenchymal stem cells
NCV	Nerve conduction velocity
NGCs	Neural guidance channels
NGF	Nerve growth factor
OE-MSCs	Olfactory mucosa stem cells
PALDE	Paracrine Signals-Loaded Extracellular Vesicle
PHEMA-MMA	Poly(2-hydroxyethyl methacrylate-co-methyl methacrylate)
PLCL	Poly(lactide-co-caprolactone)
SCI	Spinal cord injury
SFI	Sciatic functional index
SHH	Sonic hedgehog
SHH	Sonic hedgehog protein
SWCNT	Single-wall carbon nanotube
TEMPO	2,2,6,6-Tetramethylpiperidinyloxy
WD	Wallerian degeneration
